# Autologous mesenchymal stem cells for the treatment of secondary progressive multiple sclerosis: an open-label phase 2a proof-of-concept study

**DOI:** 10.1016/S1474-4422(11)70305-2

**Published:** 2012-02

**Authors:** Peter Connick, Madhan Kolappan, Charles Crawley, Daniel J Webber, Rickie Patani, Andrew W Michell, Ming-Qing Du, Shi-Lu Luan, Daniel R Altmann, Alan J Thompson, Alastair Compston, Michael A Scott, David H Miller, Siddharthan Chandran

**Affiliations:** a Department of Clinical Neurosciences, University of Cambridge, Cambridge, UK; b Anne MacLaren Laboratory for Regenerative Medicine, University of Cambridge, Cambridge, UK; c Department of Clinical Neurosciences, University of Cambridge, Cambridge, UK; d Department of Pathology, University of Cambridge, Cambridge, UK; e Department of Veterinary Medicine, University of Cambridge, Cambridge, UK; f Department of Clinical Neurosciences, University of Cambridge, Cambridge, UK; g NMR Research Unit, Institute of Neurology, Department of Neuroinflammation, University College London, London, UK; h Blood and Marrow Transplant Unit, Addenbrooke's Hospital, Cambridge, UK; i Medical Statistics Department, London School of Hygiene and Tropical Medicine, London, UK; j Department of Brain Repair and Rehabilitation, University College London, London, UK; k Centre for Clinical Brain Sciences, MRC Centre for Regenerative, Medicine University of Edinburgh, Edinburgh, UK

## Abstract

**Background:**

More than half of patients with multiple sclerosis have progressive disease characterised by accumulating disability. The absence of treatments for progressive multiple sclerosis represents a major unmet clinical need. On the basis of evidence that mesenchymal stem cells have a beneficial effect in acute and chronic animal models of multiple sclerosis, we aimed to assess the safety and efficacy of these cells as a potential neuroprotective treatment for secondary progressive multiple sclerosis.

**Methods:**

Patients with secondary progressive multiple sclerosis involving the visual pathways (expanded disability status score 5·5–6·5) were recruited from the East Anglia and north London regions of the UK. Participants received intravenous infusion of autologous bone-marrow-derived mesenchymal stem cells in this open-label study. Our primary objective was to assess feasibility and safety; we compared adverse events from up to 20 months before treatment until up to 10 months after the infusion. As a secondary objective, we chose efficacy outcomes to assess the anterior visual pathway as a model of wider disease. Masked endpoint analyses was used for electrophysiological and selected imaging outcomes. We used piecewise linear mixed models to assess the change in gradients over time at the point of intervention. This trial is registered with ClinicalTrials.gov, number NCT00395200.

**Findings:**

We isolated, expanded, characterised, and administered mesenchymal stem cells in ten patients. The mean dose was 1·6×10^6^ cells per kg bodyweight (range 1·1–2·0). One patient developed a transient rash shortly after treatment; two patients had self-limiting bacterial infections 3–4 weeks after treatment. We did not identify any serious adverse events. We noted improvement after treatment in visual acuity (difference in monthly rates of change −0·02 logMAR units, 95% CI −0·03 to −0·01; p=0·003) and visual evoked response latency (−1·33 ms, −2·44 to −0·21; p=0·020), with an increase in optic nerve area (difference in monthly rates of change 0·13 mm^2^, 0·04 to 0·22; p=0·006). We did not identify any significant effects on colour vision, visual fields, macular volume, retinal nerve fibre layer thickness, or optic nerve magnetisation transfer ratio.

**Interpretation:**

Autologous mesenchymal stem cells were safely given to patients with secondary progressive multiple sclerosis in our study. The evidence of structural, functional, and physiological improvement after treatment in some visual endpoints is suggestive of neuroprotection.

**Funding:**

Medical Research Council, Multiple Sclerosis Society of Great Britain and Northern Ireland, Evelyn Trust, NHS National Institute for Health Research, Cambridge and UCLH Biomedical Research Centres, Wellcome Trust, Raymond and Beverly Sackler Foundation, and Sir David and Isobel Walker Trust.

## Introduction

Multiple sclerosis (MS) affects more than 2 million people worldwide and is the most common non-traumatic cause of disability in young (<50 years) European adults.^1^ It is a multifocal CNS disorder that has two distinct clinical phases corresponding to inter-related pathological processes: focal inflammation that drives activity during the relapse-remitting stage and neurodegeneration that underlies progressive disease characterised by accumulating fixed disability.^2^ Although important advances in treatment to reduce relapse rate have been made in the past two decades,^3^, ^4^ no treatments are available for the roughly half of patients with MS who have progressive disease.^5^ There is therefore a great and unmet clinical need for the development of neuroprotective treatments.

Multipotent mesenchymal stromal cells are bone-marrow cells that can be expanded ex vivo and will readily differentiate into mesodermal cell derivatives.^6^ In addition to tissue engineering applications that target the repair of skeletal tissue defects,^7^ biological properties independent of differentiation suggest that mesenchymal stem cells could have a therapeutic role through strategies other than tissue replacement in diseases such as MS.^8^ These strategies include neuroprotection through paracrine effects on the CNS microenvironment, augmentation of endogenous axonal and myelin repair processes, and immune regulatory activity.^8^, ^9^ Increasing evidence shows both neuroprotection and functional improvement after infusion with mesenchymal stem cells in mouse models of relapsing-remitting and chronic MS.^10^, ^11^, ^12^, ^13^, ^14^

Clinically, mesenchymal stem cells have been used in the treatment of immune-mediated human diseases including steroid-resistant graft-versus-host disease and systemic lupus erythematosus.^15^, ^16^, ^17^ Three recent reports have also described the use of intrathecally delivered autologous mesenchymal stem cells in MS without adverse events or significant changes in general clinical outcomes (webappendix).^18^, ^19^, ^20^ However, assessment of neuroprotection in the context of MS is challenging because of clinical and pathological heterogeneity.^21^ To increase sensitivity for structural and functional treatment effects, the use of eligibility criteria that select cohorts with specific and clinically eloquent lesions, such as those of the anterior visual pathway, enables assessment of tailored and detailed outcomes.^22^, ^23^ By use of this approach, we aimed to compare safety and efficacy outcomes for patients with secondary progressive MS before and after intravenous treatment with autologous mesenchymal stem cells.

## Methods

### Participants

Between November, 2007, and August, 2010, we did an open-label phase 2a proof-of-concept study involving participants recruited from the East Anglia and north London regions of the UK (identified from MS and general neurology clinics). We screened patients for eligibility between November, 2007, and June, 2009. Eligible participants were those aged 18–65 years with clinically definite MS according to the Poser criteria,^24^ an expanded disability status scale (EDSS) score of 2·0–6·5, clinical evidence of optic nerve involvement (defined as a history of optic neuritis, Uhthoff's phenomenon, or optic atrophy on examination), abnormal visual evoked potentials from one or both eyes consistent with demyelination, a retinal nerve fibre layer thickness of at least 45 μm in one eye, a T2 lesion on MRI of the optic nerve (webappendix), and the capacity to give consent. Patients were excluded if they had a bleeding disorder, had received interferon beta or glatiramer acetate within 6 months of trial entry, or had previously used other disease modifying therapies at any point. All patients gave written informed consent before study entry and approval was obtained from the local ethics committee (Cambridgeshire 2 regional ethics committee).

### Procedures

We generated clinical-grade mesenchymal stem cells under good manufacturing practice conditions with standard operating procedures.^15^ Briefly, we separated bone-marrow mononuclear cells by density gradient centrifugation in Ficoll-Paque Premium (GE Healthcare UK Ltd, Buckinghamshire, UK). We resuspended the washed cells in phosphate-buffered saline/EDTA (Miltenyi Biotec Ltd, Surrey, UK) and cultured them in Dulbecco's modified Eagle's medium (low glucose; Invitrogen, Paisley, UK) supplemented with 10% fetal bovine serum (Hyclone; Perbio Science, Northumberland, UK), plated at a density of 1×10^8^ cells per cell factory (Nunc, Thermo Scientific, Northumberland, UK). Near confluent cultures (>80%) were treated with 0·25% trypsin-EDTA (Invitrogen) and replated at 3·5×10^6^ cells per cell factory. Mesenchymal stem cells were harvested and cryopreserved in 4·5% human albumin solution (Bio Products Laboratory, Hertfordshire, UK) with dimethyl sulphoxide (Origen Biomedical Inc, Helsingborg, Sweden) at a final concentration of 10%. Mesenchymal stem cells were then characterised in accordance with International Society of Cellular Therapy recommendations.^25^ Briefly, this included evidence of trilineage differentiation potential (adipocyte, chondrocyte, osteocyte) and flow cytometry to confirm expression of CD73, CD90, and CD105 surface molecules (>95%) and absence of CD34, CD45, CD14, and CD3 (<2%). Release criteria for clinical use included absence of contamination by pathogens (as documented by aerobic and anaerobic cultures and mycoplasma testing), and lack of any genomic copy number changes as assessed with 1-Mb-resolution bacterial artificial chromosome array comparative genomic hybridisation.^26^

We administered autologous mesenchymal stem cells intravenously to patients with secondary progressive MS. Administration of the cells was done as a day-case procedure. To reduce type I hypersensitivity reactions, premedication with 10 mg chlorpheniramine, 100 mg hydrocortisone, and 10 mg metoclopramide was given 30 min before administration of the cells. Cryopreserved cells were thawed (≤4 min) and immediately infused over 15 min through a peripheral venous cannula. After administration of cell suspensions, we infused normal saline (500 mL) over 4 h.

Our primary objective was to assess feasibility and safety; our secondary objectives were to assess efficacy on clinical, electrophysiological, and structural outcomes, in addition to providing information on the mechanism of any recorded effect. We used a sentinel lesion approach based on the diseased anterior visual pathway to increase power to detect treatment effects,^23^ and we used a pretest–post-test design to compare adverse events and efficacy measures before and after the intervention. We assessed participants at 3–6 month intervals for at least 12 months before and 6 months after treatment (webappendix). Assessment at each timepoint was split into two visits with a gap of less than 2 weeks; clinical assessment and visual evoked responses were done in Cambridge, UK, and MRI, optical coherence tomography, and neuro-ophthalmological assessments were done at the University College London Institute of Neurology (London, UK). Clinical assessment involved neurological and medical history with recording of adverse events and scores on the EDSS, MS functional composite (MSFC), Addenbrooke's cognitive examination revised, 29-item MS impact scale, and Beck depression inventory II. Whole and central field checkerboard pattern-reversal visual evoked responses were recorded with reversal achromatic checks subtending 60′ at the eye. Neuro-opthalmological assessment included visual acuity with a retroilluminated early treatment diabetic retinopathy study chart, contrast acuity with retroilluminated Sloan charts, colour vision with the Farnsworth–Munsell 100-hue test, and visual field assessment by automated static perimetry (Humphrey field analyser, 30-2 protocol). Optical coherence tomography images were acquired by a single operator (MK) with a time domain optical coherence tomograph (Stratus OCT Model 3000; Carl Zeiss Meditec, Dublin, CA, USA).^23^

MRI images were acquired with a Magnetom 3·0 T Tim Trio scanner (Siemens, Erlangen, Germany) with a 12-element receiver head coil. Several MRI-based measures were assessed: optic nerve cross-sectional area; optic nerve diffusion tensor imaging measures of fractional anisotropy, mean diffusivity, axial diffusivity, and radial diffusivity; optic nerve magnetisation transfer ratio; whole-brain T2 lesion volume; whole-brain T1 hypointense lesion volume; whole-brain magnetisation transfer ratio; brain T1 hypointense lesion magnetisation transfer ratio; and brain T2 lesion magnetisation transfer ratio.^23^ Intersessional stability of imaging measures was confirmed by contemporary assessment of ten locally recruited healthy volunteers.

Visual evoked responses, optic nerve area, optic nerve magnetisation transfer ratio, and optic nerve diffusion tensor imaging based outcomes were assessed by a single observer (MK) from whom participant status (before or after treatment) was masked. Lesional analysis was done after image acquisition at each visit. Brain volume and whole-brain magnetisation transfer ratio were done with automated methods with minimal manual corrections.

### Statistical analysis

We used piecewise linear mixed models to assess,^27^ for a given measure, the change in gradient over time at the point of intervention; the given measure was the response variable, with the time from intervention and the time multiplied by an after-intervention interaction term as the two predictors. Such models allow estimation of the gradients before and after intervention, and of the gradient change with its statistical significance. For analyses of data involving separate values for each eye over time, we added an additional level to the model with individual eyes as levels within participants. For the EDSS score, although the before and after gradients were estimated as above, the test of gradient change used the non-parametric Wilcoxon sign rank test to compare the two ratios: change in EDSS score before or time interval before versus change in score after or time interval after. There was no evidence of deviation from model assumptions. In particular there was no evidence for non-normality or heteroscedasticity of residuals, or evidence against linearity assumptions. All of the reported mixed models achieved convergence with estimates for both the variance components and the fixed effects. Unrecordable visual evoked responses, related to severe dysfunction due to disease, were represented by amplitude values of 0 μV and latency values of 180 ms (the maximum recorded during our study). Analyses were done with Stata SE (versions 9.2 and 11). Power calculations could not be done before the study because of the lack of information from previous studies on potential effect sizes. This trial is registered with ClinicalTrials.gov, number NCT00395200.

### Role of the funding source

The sponsors of the study had no role in study design, data collection, data analysis, data interpretation, or writing of the report. The corresponding author had full access to all the data in the study and had final responsibility for the decision to submit for publication.

## Results

**Figure 1:**
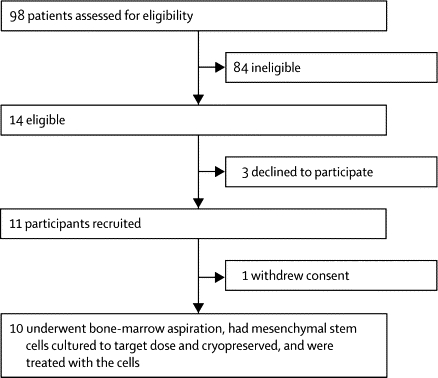
Study profile

Figure 1 shows the study profile and table 1 the participants' characteristics. All participants had secondary progressive MS,^28^ with clinical and electrophysiological evidence of optic nerve involvement. In the 2–26 years before recruitment, nine patients had clinical optic neuritis (three bilateral) and one had Uhthoff's phenomenon. Two patients described a single clinical relapse event in the pretreatment phase, neither of which involved the anterior visual pathway. One patient had been previously treated with disease modifying therapy (interferon beta for 1 year, with treatment discontinued owing to disease progression 2 years before recruitment into our trial).

**Table 1:** Participants' characteristics

	**Measure at recruitment**
Number of participants	10
Sex ratio (men:women)	7:3
Age (years)	48·8 (4·1; 40–53)
Duration of multiple sclerosis (years)	14·4 (7·9; 5–26)
Expanded disability status score	6·1 (0·3; 5·5–6·5)
Time since last clinical episode of optic neuritis (years)*	11·9 (8·2; 2–26)

Data are n or mean (SD; range).

* Nine participants.

We successfully isolated and cultured mesenchymal stem cells to the target dose from all bone-marrow aspirates (mean total cultured dose 2·0×10^6^ cells per kg, range 1·1×10^6^–3·7×10^6^). Mean culture duration was 24 days (20–30). Patients received a single infusion of autologous cells after monitoring for a mean of 17·3 months (14·1–20·9) during the pretreatment phase. The mean administered dose was 1·6×10^6^ cells per kg bodyweight (1·1×10^6^–2·0×10^6^); mean volume of cell suspensions was 167·2 mL (range 89–246). We did not record any adverse events during infusion. One patient developed a macular rash over the anterior chest at about 3 h after the start of infusion that resolved spontaneously over 12 h; a further patient described scalp pruritus beginning 1 week after treatment and resolving spontaneously 2 weeks later. Two patients had infections: a self-limiting upper-respiratory tract infection 3 weeks after infusion (not requiring treatment) and an *Escherichia coli* urinary-tract infection 4 weeks after infusion (treated with oral antibiotics). Results of weekly blood testing of clinical chemistry, haematology, and immunology during the 4 weeks after infusion was unremarkable. Compared with pretreatment titres, no changes were evident in the post-treatment period for T-cell subset counts (CD3, CD4, CD8, CD19, and CD56) or humoral immunity assessed by titres to common antigens (mumps, measles, rubella, varicella zoster, tetanus, *Haemophilus influenzae* type B, and pneumococcal antigens 1, 3, 4, 5, 6B, 7F, 8, 14, 18C, 19A, 19F, and 23F). We did not identify any delayed adverse events during the post-treatment phase (mean 7·0 months, 5·8–10·2).

**Figure 2:**
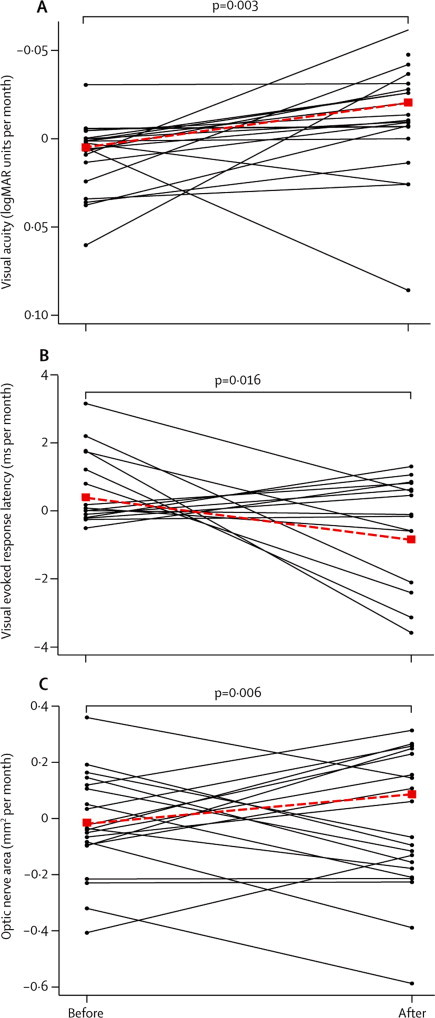
Change in visual function, visual evoked response amplitude, and optic nerve area Paired monthly estimated rates of change in log of minimum angle of resolution (logMAR) visual acuity, whole-field visual evoked response latency, and optic nerve area are shown for individual patients before and after treatment connected by solid lines. Pretreatment and post-treatment mean rates of change are also shown connected with a dashed line. Significance tests are shown for a difference between mean rates of change before and after treatment.

After treatment, there was an improvement in log of minimum angle of resolution (logMAR) visual acuity (figure 2, table 2) and low contrast visual acuity (table 2, webappendix). No significant changes were evident in colour vision or visual fields. Physiological measures showed a post-treatment reduction in visual evoked response latency and an increase in visual evoked response amplitude; imaging measures showed an increase in optic nerve area after treatment (figure 2, table 2). No change was evident in macular volume, retinal nerve fibre layer thickness, or optic nerve magnetisation transfer ratio.

**Table 2:** Efficacy outcomes

	**Rate of change**		**Difference in rate of change after treatment (95% CI)**	**p value**
	Before treatment	After treatment		
**Vision**				
Visual acuity (logMAR)	0·0050	−0·0207	−0·0205 (−0·0325 to −0·0085)	0·003
25% contrast acuity (logMAR)	0·0022	−0·0207	−0·0202 (−0·0330 to −0·0073)	0·011
5% contrast acuity (logMAR)	0·0083	−0·0372	−0·0371 (−0·0560 to −0·0181)	0·001
1·25% contrast acuity (logMAR)	0·0063	−0·0370	−0·0369 (−0·0552 to −0·0185)	0·001
Colour vision (Farnsworth–Munsell 100-hue test √total error score)	0·1017	−0·0975	−0·1011 (−0·2567 to 0·0544)	0·070
Visual field (mean deviance)	0·0395	0·00311	0·0192 (−0·1062 to 0·1445)	0·893
Full field visual evoked response latency (ms)	0·4843	−0·8438	−1·3280 (−2·4447 to −0·2114)	0·020
Full field visual evoked response amplitude (μV)	−0·1084	0·1503	0·2587 (0·0705 to 0·4469)	0·007
Macular volume (mm^3^)	0·0002	0·0041	0·0040 (−0·0135 to 0·0214)	0·654
Retinal nerve fibre layer thickness (μm)	−0·0052	0·0474	0·0527 (−0·3533 to 0·4586)	0·799
Optic nerve area (mm^2^)	−0·0216	0·1046	0·1262 (0·0368 to 0·2155)	0·006
Optic nerve magnetisation transfer ratio (pu)	0·0656	0·0529	0·0529 (−0·1271 to 0·2328)	0·565
**General**				
Expanded disability status scale	0·0257	−0·0012	−0·0269 (−0·0431 to −0·0107)	0·028
Multiple sclerosis functional composite (*Z* score)	−0·0217	0·0141	0·0359 (−0·0275 to 0·0992)	0·267
Addenbrooke's cognitive examination (revised)	0·0492	0·2690	0·2198 (−0·1343 to 0·5739)	0·224
29-item multiple sclerosis impact scale	−0·3710	−0·5152	−0·1443 (−2·0865 to 1·7979)	0·884
Beck depression inventory II	0·0965	−0·2663	−0·3628 (−0·9378 to 0·2121)	0·216
T1 lesion volume (mm^3^)	204·35	−60·73	−265·08 (−574·85 to 44·69)	0·094
T1 lesion magnetisation transfer ratio (pu)	−0·1867	0·5791	0·7659 (−0·1389 to 1·6706)	0·097
T2 lesion volume (mm^3^)	155·89	20·90	−134·98 (−579·64 to 309·67)	0·552
T2 lesion magnetisation transfer ratio (pu)	−0·1738	0·3859	0·5597 (−0·2703 to 1·3896)	0·186
Total brain volume (%)	−0·0880	−0·1470	−0·0590 (−0·1434 to 0·0254)	0·171

Data are units per month unless otherwise stated. MAR=minimum angle of resolution. pu=percent units.

There was reduction after treatment in general disability progression measured by EDSS (table 2). We did not identify a change in the MSFC or in measures of depression, cognition, and self-reported effect of MS on daily living. T1 hypointense lesion volume decreased after treatment and magnetisation transfer ratio increased, but these changes were not statistically significant. We did not identify any changes in the rate of T2 lesion accumulation or general brain atrophy after treatment.

## Discussion

Our proof-of-concept study provides evidence that an intervention might affect the disease course in progressive MS. Specifically, we show that after intravenous administration of autologous mesenchymal stem cells, patients with secondary progressive disease improved on measures of visual function, physiology, and structure without evidence of significant adverse events. Improvements in visual acuity and contrast sensitivity after treatment were accompanied by changes in masked outcome measures, as a reduction in visual evoked response latency, increase in visual evoked response amplitude, and an increase in optic nerve area. General disability progression measured by EDSS was also reduced after treatment.

Despite recent major advances in immunomodulatory therapies, there are no treatments to slow, stop, or reverse the accumulation of fixed disability in secondary progressive MS. This relates in part to the complex and incompletely understood biology of progression. Furthermore, assessing neuroprotective therapies in MS presents a substantial challenge because of the variability in disease features and course, combined with insensitivity of generic clinical outcomes. We therefore adopted a sentinel lesion approach based on a detailed assessment of the anterior visual pathway as a model of wider processes. We chose the anterior visual pathway because of convergence of reliable and validated outcomes for clinical function, physiology, and structure.^22^ Nevertheless, because of wide variation between individuals in the rate of disease progression, a further challenge in testing advanced therapies such as cell-based interventions is to design early stage trials that achieve adequate power. On this basis, we used a pretest–post-test design to increase effect size and therefore increase statistical power by 40–80%.^23^ The limitations of this approach are that change evident after treatment cannot be attributed exclusively to the effects of treatment since factors we did not record might also contribute. Changes identified after treatment therefore need to be confirmed as treatment effects by replication in trials with random allocation between comparator groups. Such trials require substantial investment, and feasibility, safety, and effect-size-defining studies such as ours therefore have a key role in informing decisions about whether further studies are justifiable and how they should be designed. Further limitations of our study include the small cohort size and lack of masking for clinical outcomes. There is also risk of type I error due to multiple statistical comparisons; our results should therefore be regarded as hypothesis generating and will need confirmation in future studies. Nevertheless, interpretation of post-treatment changes is aided by masked electrophysiological and imaging outcomes that are probably resistant to observer bias or placebo effects. Moreover, unlike designs in which treatment is started immediately after recruitment, post-treatment change in our study is robust to regression to the mean because of our prolonged pretreatment assessment phase.

We do not know the precise mechanism by which mesenchymal stem cells might act in our study. However, the findings from our masked analyses showed an increase in optic nerve area and reduction in visual evoked response latency that are consistent with a neuroprotective effect because of the promotion of myelin repair. Our findings of a possible reduction in brain T1 hypointense lesion volume and an increase in brain T1 lesion magnetisation transfer ratio provide indirect support for this idea.^29^, ^30^ Remyelination is the regenerative process by which myelin sheaths are restored to demyelinated axons and the failure of remyelination is implicated in the neuronal and axonal loss that underlies progressive disability.^31^ Although our study was not designed specifically to address the effects of intervention on inflammatory MRI metrics, we did not identify any change in the rate of T2 lesion accumulation after treatment. Similarly, the lack of effect on optical coherence tomography measures after treatment supports a view that structural change in unmyelinated axons was not a significant factor. The lack of significant change in optic nerve magnetisation transfer ratio measures suggests that the clinical and neurophysiological improvements might relate to diffuse tissue repair in the diseased anterior visual pathways or to our small study size and technical limitations, in view of the restricted previous experience of optic nerve magnetisation transfer ratio at 3 T. Taken together our findings are consistent with a growing body of published work in acute and chronic models of MS showing neuroprotective effects of mesenchymal stem cells independent of directed differentiation or cell replacement (panel).^8^, ^9^ Central or peripheral mechanisms that might explain these results include immunoregulation, and modification of the cellular environment causing trophic or anti-inflammatory effects. Furthermore, recent studies suggest that mesenchymal stem cells can promote the endogenous CNS repair processes of oligodendrogenesis and remyelination,^32^, ^33^ raising the possibility that remyelination underlies the evidence for neuroprotection in our study.

Panel **Research in context**

**Systematic review**

We searched Medline (1950 to August, 2011), Embase (1980 to August, 2011), and the Cochrane Central Register of Controlled Trials (The Cochrane Library issue 4, 2011) with the terms “multiple sclerosis” and “mesenchymal stem cells” for clinical trials published up to August, 2011, that report the effect of mesenchymal stem cells on the rate of disease progression in secondary progressive multiple sclerosis. We did not limit our search by language. We identified three published trials in which autologous ex-vivo expanded mesenchymal stem cells were administered intrathecally,^18^ intrathecally and intracisternally,^19^ or intrathecally and intravenously (webappendix).^20^

**Interpretation**

Consistent with previous studies, our findings show that intravenous administration of autologous mesenchymal stem cells to patients with secondary progressive multiple sclerosis is feasible and safe. Our findings also suggest structural, functional, and physiological improvement after treatment consistent with neuroprotection.

## Web Extra Material

Supplementary webappendix
