# Supplementary material for: Autologous mesenchymal stem cells for the treatment of secondary progressive multiple sclerosis: an open-label phase 2a proof-of-concept study
Source: Lancet Neurol. 2012 Feb;11(2):150–6. doi: 10.1016/S1474-4422(11)70305-2 (PMC3279697; doi:10.1016/S1474-4422(11)70305-2)
Supplement: Supplementary webappendix [file mmc1.pdf]

THE LANCET Neurology

Supplementary webappendix

This webappendix formed part of the original submission and has been peer reviewed. We post it as supplied by the authors.

Supplement to: Connick P, Kolappan M, Crawley C, et al. Autologous mesenchymal stem cells for the treatment of secondary progressive multiple sclerosis: an open-label phase 2a proof-of-concept study. *Lancet Neurol* 2012; published online Jan 10. DOI:10.1016/S1474-4422(11)70305-2.

Supplementary table 1: Existing studies using mesenchymal stem cells in multiple sclerosis

Author	Description
Mohyeddin et al. [19]	Ten patients with RRMS were administered autologous MSCs intrathecally, and followed for a mean of 19 months by clinical and imaging assessment. No significant changes were seen in clinical or imaging outcomes.
Yamout et al.	Ten patients with SPMS were administered autologous MSCs intrathecally, and followed for 12 months by clinical and imaging assessment. Clinical assessment suggested possible improvement but no significant change was seen on imaging outcomes.
Karussis et al.	Fifteen patients with SPMS were administered autologous MSCs both intrathecally and intravenousy, and followed for 6 months by clinical and imaging assessments. No significant adverse events, or changes on clinical or imaging outcomes were seen.

Supplementary table 2: Assessment schedule

	**Pre-treatment**								**Post-treatment**							
**Time-line**	**-12 months**		**-9 months**		**-6 months**		**0 months**		**1 week**	**2 weeks**	**3 weeks**	**4 weeks**	**3 months**		**6 months**	
**Visit number**	**1**	**2**	**3**	**4**	**5**	**6**	**7**	**8**	**9**	**10**	**11**	**12**	**13**	**14**	**15**	**16**
EDSS	X		X		X		X						X		X	
MSFC	X		X		X		X						X		X	
MSIS-29	X		X		X		X						X		X	
BDI-II	X		X		X		X						X		X	
ACE-R	X		X		X		X						X		X	
VEP	X		X		X		X						X		X	
LogMAR visual acuity		X		X		X		X						X		X
Farnsworth Munsell 100 Hue test		X		X		X		X						X		X
Humphrey Visual perimetry		X		X		X		X						X		X
OCT		X		X		X		X						X		X
MRI Optic Nerve		X		X		X		X						X		X
MRI Brain		X		X		X		X						X		X
MRI Brain (DTI sequence)				X												X
Clinical chemistry	X						X		X	X	X	X				
FBC, ESR	X						X		X	X	X	X				
Immuno-globulins							X		X	X	X	X				
C3 & C4																
T cell subsets							X		X	X	X	X				
Antibody titres							X		X	X	X	X				
PT & APTT	X						X		X	X	X	X				

EDSS = expanded disability status scale; MSFC = multiple sclerosis functional composite; BDI-II = Beck depression inventory II; ACE-R = Addenbrooke's cognitive examination (revised); VEP = visual evoked potential; OCT = optical coherence tomography; DTI = diffusion tensor imaging; Clinical chemistry = serum urea and

electrolytes, liver function tests, serum calcium, glucose, & thyroid function; FBC = full blood count; ESR = erythrocyte sedimentation rate; Immunoglobulins = serum IgG, IgA, & IgM; C3 & C4 = serum complement component 3 and serum complement component 4; T-cell subsets = differential count for CD3, CD4, CD8, CD19, & CD56; Antibody titres = serum titres for Haemophilus influenzae type-b IgG, tetanus IgG, rubella IgG, varicella zoster virus IgG, mumps IgG, measles IgG, and pneumococcal IgG for serotypes 1, 3, 4, 5, 6A, 6B, 7F, 9V, 14, 18C, 19A, 19F, & 23F; PT = prothrombin time; APTT = accelerated partial thromboplastin time.

Supplementary table 3: Change in key visual outcomes by individual patients

Subject ID	Optic nerve ID	Outcome Measure (units)	Pre-treatment -12 months	Pre-treatment -9 months	Pre-treatment -6 months	Pre-treatment 0 months	Post-treatment +3 months	Post-treatment +6 months
A	1	Visual acuity (LogMAR)	-0.14	0.1	0.04	-0.06	0	-0.06
		25% contrast acuity (logMAR)	0.1	0.24	0.22	0	0.02	0.06
		5% contrast acuity (logMAR)	0.6	0.56	0.48	0.4	0.4	0.56
		1.25% contrast acuity (logMAR)	0.76	0.76	0.76	0.76	0.7	0.78
		Full field VER latency (ms)	138	138	142.8	140	139	146.1
		Full field VER amplitude (μV)	9	9	8	6	7	4.8
		Optic nerve area (mm^2)	9.55	9.58	9.025	8.6	10.93	9.78
	2	Visual acuity (LogMAR)	-0.1	0	0	-0.06	-0.06	-0.06
		25% contrast acuity (logMAR)	0	0.06	0.1	0.04	0	0.04
		5% contrast acuity (logMAR)	0.3	0.4	0.3	0.28	0.18	0.28
		1.25% contrast acuity (logMAR)	0.54	0.74	0.6	0.56	0.5	0.66
		Full field VER latency (ms)	120	120	124	118	121	123
		Full field VER amplitude (μV)	8	8	5.8	2	9	6.1
		Optic nerve area (mm^2)	10.3	10.26	10.25	10.9	12.15	11.53
B	3	Visual acuity (LogMAR)	0.08	0.64	0.22	0.54	0.16	0.04
		25% contrast acuity (logMAR)	0.32	0.68	0.3	0.58	0.2	0.2
		5% contrast acuity (logMAR)	0.7	1.4	0.86	1.4	0.54	0.6
		1.25% contrast acuity (logMAR)	1.4	1.7	1.7	1.7	1.3	1.38
		Full field VER latency (ms)	133	135	-	NR	136.5	138.9
		Full field VER amplitude (μV)	4	6	-	NR	3.6	3.1
		Optic nerve area (mm^2)	10.74	8.05	8.05	-	8.2	7.825
	4	Visual acuity (LogMAR)	0.1	0.2	0.2	0.2	0.08	0
		25% contrast acuity (logMAR)	0.3	0.46	0.36	0.3	0.28	0.08
		5% contrast acuity (logMAR)	0.66	0.74	0.8	0.7	0.64	0.56
		1.25% contrast acuity (logMAR)	1.4	1.7	1.7	1.7	1.24	1.28
		Full field VER latency (ms)	136	140	-	133.5	133.5	138.9
		Full field VER amplitude (μV)	5	7	-	6.5	6.7	5.3
		Optic nerve area (mm^2)	7.86	6.4	5.75	-	7.63	5.95
C	5	Visual acuity (LogMAR)	0.02	0.16	0.02	0.14	0.1	0.04
		25% contrast acuity (logMAR)	0.2	0.14	0.16	0.3	0.22	0.16
		5% contrast acuity (logMAR)	0.5	0.68	0.46	0.48	0.52	0.4
		1.25% contrast acuity (logMAR)	0.8	1.4	0.82	0.92	0.96	0.64
		Full field VER latency (ms)	119	118	113.7	116	108.3	115.2
		Full field VER amplitude (μV)	4	4.6	5.8	3.8	3.9	6.1
		Optic nerve area (mm^2)	9.4	8.85	8.525	6.475	9.6	8.3
	6	Visual acuity (LogMAR)	0.04	0.18	0	0.02	0.06	0.02
		25% contrast acuity (logMAR)	0.04	0.18	0	0.02	0.06	0.02
		5% contrast acuity (logMAR)	0.5	0.6	0.56	0.42	0.52	0.26
		1.25% contrast acuity (logMAR)	0.94	1.4	0.86	0.72	1.08	0.62
		Full field VER latency (ms)	119	118	118.2	120	112.2	115.5
		Full field VER amplitude (μV)	6	4.6	6.8	2.7	4.3	5.4
		Optic nerve area (mm^2)	8.95	7.96	6.8	6.225	8.76	7.53

D	7	Visual acuity (LogMAR)	0.16	0.3	-	0.1	0.06	0.02
		25% contrast acuity (logMAR)	0.3	0.54	-	0.14	0.16	0.1
		5% contrast acuity (logMAR)	0.6	0.78	-	0.36	0.44	0.38
		1.25% contrast acuity (logMAR)	0.88	0.92	-	0.76	0.76	0.68
		Full field VER latency (ms)	123	126	124.5	123	120	124
		Full field VER amplitude (μV)	5	4	3.5	2.3	5	4
		Optic nerve area (mm²)	9.166	8.983	-	8.816	8.14	8.82
	8	Visual acuity (LogMAR)	0	0.02	-	-0.08	0.06	0.04
		25% contrast acuity (logMAR)	0	0.12	-	0.02	0.14	0.02
		5% contrast acuity (logMAR)	0.3	0.46	-	0.34	0.5	0.34
		1.25% contrast acuity (logMAR)	0.2	0.8	-	0.72	0.98	0.62
		Full field VER latency (ms)	123	143	126	123.6	158	139
		Full field VER amplitude (μV)	3	3	2	1.2	3	4
		Optic nerve area (mm²)	8.54	8.34	-	7.8	8.28	8.74
E	9	Visual acuity (LogMAR)	0.2	0.24	-	0.2	0.08	0.14
		25% contrast acuity (logMAR)	0.5	0.42	-	0.54	0.16	0.24
		5% contrast acuity (logMAR)	0.8	0.7	-	0.92	0.7	0.52
		1.25% contrast acuity (logMAR)	1.7	1.7	-	1.7	0.92	0.88
		Full field VER latency (ms)	151	NR	NR	NR	156	-
		Full field VER amplitude (μV)	3.2	NR	NR	NR	4	-
		Optic nerve area (mm²)	5.25	6.325	-	6.975	6.725	8.675
	10	Visual acuity (LogMAR)	0.24	0.24	-	0.22	0.2	0.12
		25% contrast acuity (logMAR)	0.34	0.3	-	0.22	0.36	0.36
		5% contrast acuity (logMAR)	0.74	0.6	-	0.6	0.64	0.6
		1.25% contrast acuity (logMAR)	0.88	0.9	-	0.94	0.98	0.88
		Full field VER latency (ms)	107	NR	NR	NR	NR	-
		Full field VER amplitude (μV)	0.9	NR	NR	NR	NR	-
		Optic nerve area (mm²)	7.05	7.625	-	8.95	7.86	8.275
F	11	Visual acuity (LogMAR)	0	-0.16	-	0.02	-0.26	-0.04
		25% contrast acuity (logMAR)	0	0.02	-	0	0.04	0.09
		5% contrast acuity (logMAR)	0.36	0.28	-	0.24	0.3	0.3
		1.25% contrast acuity (logMAR)	0.72	0.5	-	0.5	0.52	0.56
		Full field VER latency (ms)	144	129	-	133.3	131	140.7
		Full field VER amplitude (μV)	3	4	-	4.1	5	3.4
		Optic nerve area (mm²)	6.64	7.375	-	8.08	7.775	8.58
	12	Visual acuity (LogMAR)	0.4	-0.1	-	-0.04	0.02	-0.06
		25% contrast acuity (logMAR)	0.6	0.04	-	0.1	0.1	0.1
		5% contrast acuity (logMAR)	0.36	0.22	-	0.2	0.3	0.22
		1.25% contrast acuity (logMAR)	0.6	0.42	-	0.5	0.54	0.54
		Full field VER latency (ms)	106	105	-	107.1	102	104.1
		Full field VER amplitude (μV)	4	3	-	4.5	3	2.4
		Optic nerve area (mm²)	8.45	8.84	-	10.1	10.9	10.5
G	13	Visual acuity (LogMAR)	0.18	0.24	-	0.78	0.14	0.04
		25% contrast acuity (logMAR)	0.38	0.36	-	1.04	0.2	0.14
		5% contrast acuity (logMAR)	0.7	0.62	-	1.7	0.4	0.26
		1.25% contrast acuity (logMAR)	0.8	0.96	-	1.7	0.6	0.4
		Full field VER latency (ms)	114	123.6	-	130.5	120.9	117.6
		Full field VER amplitude (μV)	5	5.6	-	1.8	7.2	5.1

		Optic nerve area (mm²)	9.26	8.2	-	8.6	8.3	9.025
	14	Visual acuity (LogMAR)	0.4	0.38	-	0.76	0.12	0.1
		25% contrast acuity (logMAR)	0.64	0.58	-	0.98	0.2	0.18
		5% contrast acuity (logMAR)	0.8	0.76	-	1.7	0.36	0.36
		1.25% contrast acuity (logMAR)	1	1	-	1.7	0.56	0.54
		Full field VER latency (ms)						
		Full field VER amplitude (µV)						
		Optic nerve area (mm²)	7.8	8.23	-	7.266	7.46	7.625
H	15	Visual acuity (LogMAR)	0.66	0.86	-	0.74	0.86	0.96
		25% contrast acuity (logMAR)	0.8	0.88	-	1	0.98	0.98
		5% contrast acuity (logMAR)	1.7	1.7	-	1.7	1.58	1.48
		1.25% contrast acuity (logMAR)	1.7	1.7	-	1.7	1.7	1.7
		Full field VER latency (ms)	NR	NR	-	NR	NR	NR
		Full field VER amplitude (µV)	NR	NR	-	NR	NR	NR
		Optic nerve area (mm²)	7.76	7.475	-	8.025	7.2	8.2
	16	Visual acuity (LogMAR)	0.28	0.48	-	0.68	0.56	0.66
		25% contrast acuity (logMAR)	0.6	0.6	-	0.7	0.74	0.86
		5% contrast acuity (logMAR)	0.84	0.92	-	1.7	1.48	1.26
		1.25% contrast acuity (logMAR)	1.7	1.7	-	1.7	1.7	1.7
		Full field VER latency (ms)	111	NR	-	NR	NR	NR
		Full field VER amplitude (µV)	4	NR	-	NR	NR	NR
		Optic nerve area (mm²)	8.73	8.55	-	7.7	8.1	9.067
I	17	Visual acuity (LogMAR)	0.04	0.06	-	0.04	0.04	-0.04
		25% contrast acuity (logMAR)	0.02	0.14	-	0.12	0.08	0.04
		5% contrast acuity (logMAR)	0.36	0.42	-	0.42	0.38	0.28
		1.25% contrast acuity (logMAR)	0.7	0.56	-	0.78	0.76	0.66
		Full field VER latency (ms)	133	142.2	139	134.1	133.8	142
		Full field VER amplitude (µV)	8	7.9	3.5	1.8	3.8	5
		Optic nerve area (mm²)	7.65	7.125	-	7.275	8.276	7.725
	18	Visual acuity (LogMAR)	0.06	0.14	-	0.08	0.06	-0.02
		25% contrast acuity (logMAR)	0.18	0.16	-	0.2	0.16	0.16
		5% contrast acuity (logMAR)	0.46	0.4	-	0.52	0.48	0.46
		1.25% contrast acuity (logMAR)	0.92	0.78	-	0.98	0.88	0.88
		Full field VER latency (ms)	144	156.9	148	152.7	133.8	164
		Full field VER amplitude (µV)	5	4.5	4.3	2.2	4	3
		Optic nerve area (mm²)	8.125	7.3	-	7.2	6.33	6.66
J	19	Visual acuity (LogMAR)	0.18	0.18	-	0.26	0.2	0.02
		25% contrast acuity (logMAR)	0.24	0.3	-	0.32	0.3	0.18
		5% contrast acuity (logMAR)	0.5	0.48	-	0.52	0.44	0.36
		1.25% contrast acuity (logMAR)	0.7	0.66	-	0.78	0.62	0.58
		Full field VER latency (ms)	164	163.8	-	NR	168.3	-
		Full field VER amplitude (µV)	4	3.7	-	NR	1.3	-
		Optic nerve area (mm²)	8.1	8.53	-	9.63	9.925	9.73
	20	Visual acuity (LogMAR)	0.14	0.14	-	0.46	0.14	0.18
		25% contrast acuity (logMAR)	0.44	0.28	-	0.38	0.34	0.16
		5% contrast acuity (logMAR)	0.6	0.4	-	0.58	0.4	0.38
		1.25% contrast acuity (logMAR)	0.74	0.66	-	0.76	0.56	0.6

Full field VER latency (ms)	154	156.9	-	NR	171	-
Full field VER amplitude (μV)	3	2.5	-	NR	0.7	-
Optic nerve area (mm^2)	8.175	9.1	-	9.275	9.2	8.86

NR = No response; - = not performed.

Supplementary figure 1: Coronal T2-weighted MRI of the optic nerves

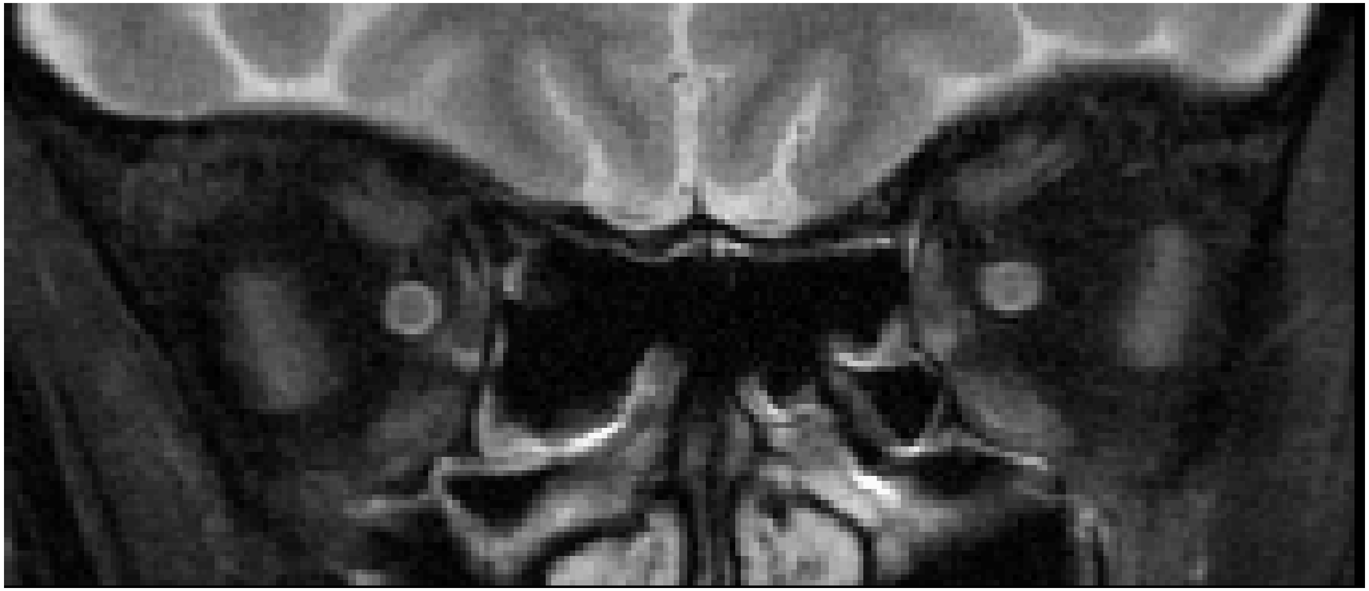

Hyperintensity of the left optic nerve is shown on coronal T2-weighted MR imaging.

Supplementary figure 2A: change in visual acuity over time

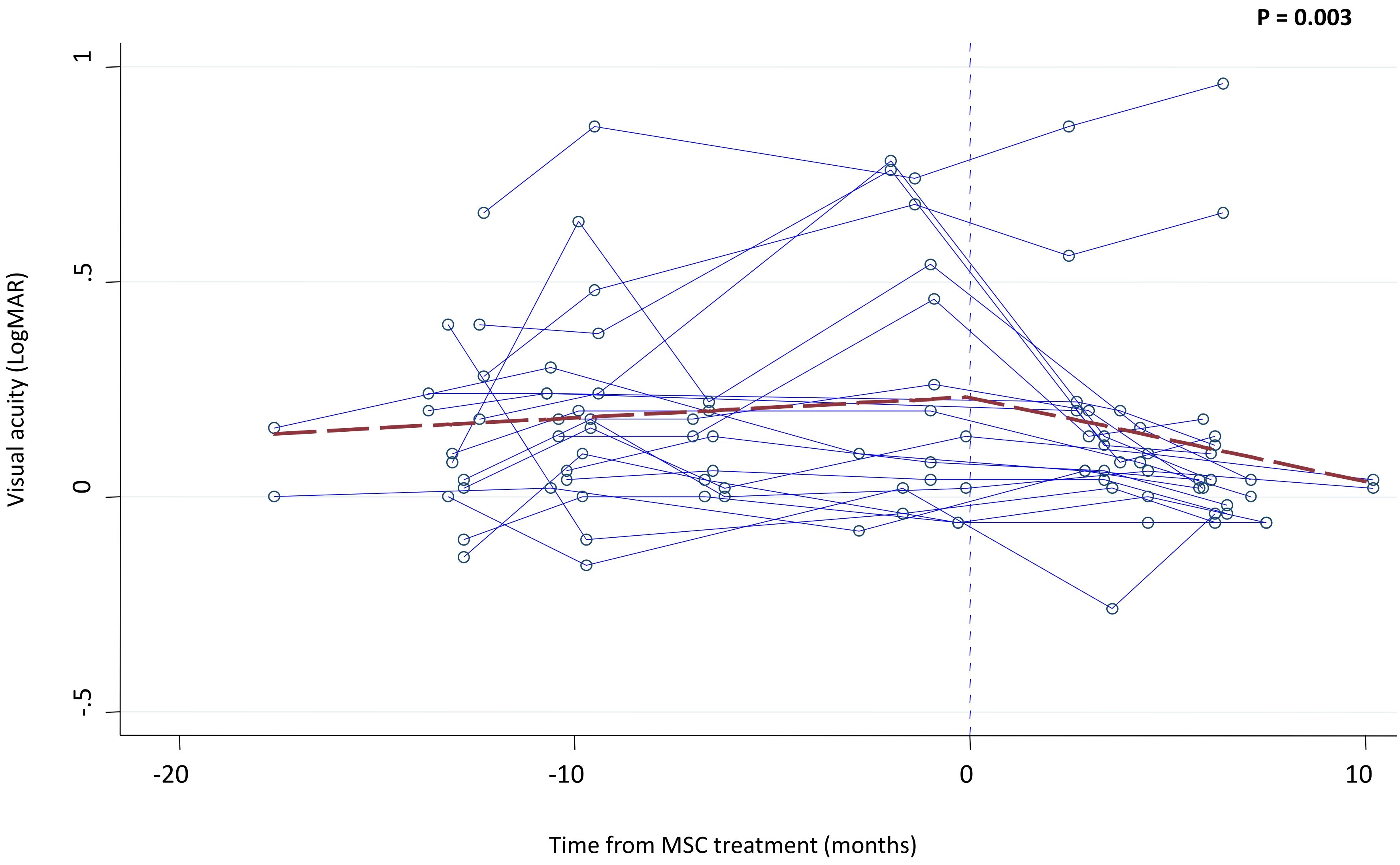

Change in visual acuity is shown for individual patients before and after treatment connected by solid lines. Pre- and post-treatment average rates of change are also shown connected in red dash. The significance test are shown is for a change in the gradient at the time of intervention.

Supplementary figure 2B: change in 25% contrast acuity over time

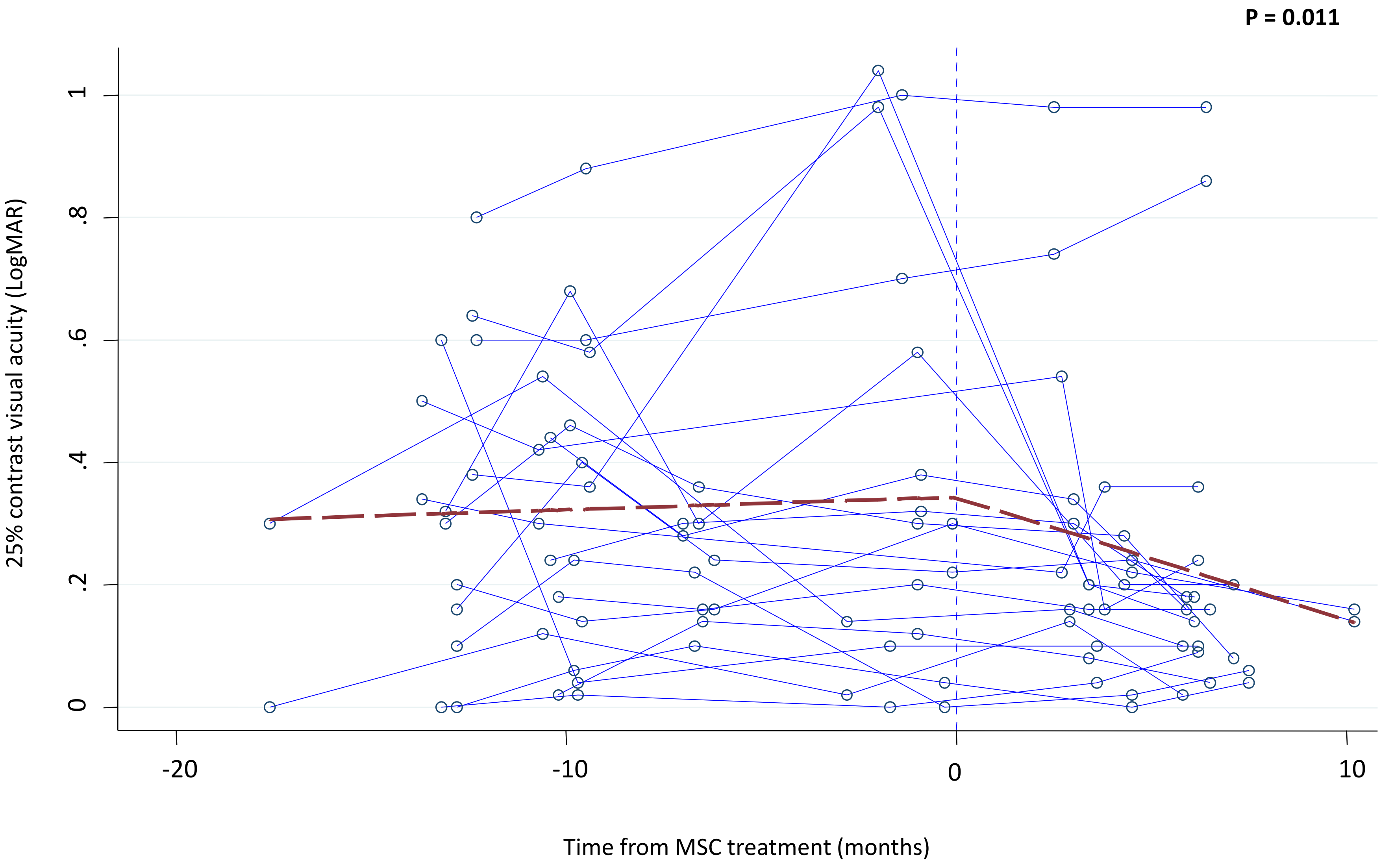

Change in 25% contrast visual acuity is shown for individual patients before and after treatment connected by solid lines. Pre- and post-treatment average rates of change are also shown connected in red dash. The significance test are shown is for a change in the gradient at the time of intervention.

Supplementary figure 2C: change in 5% contrast acuity over time

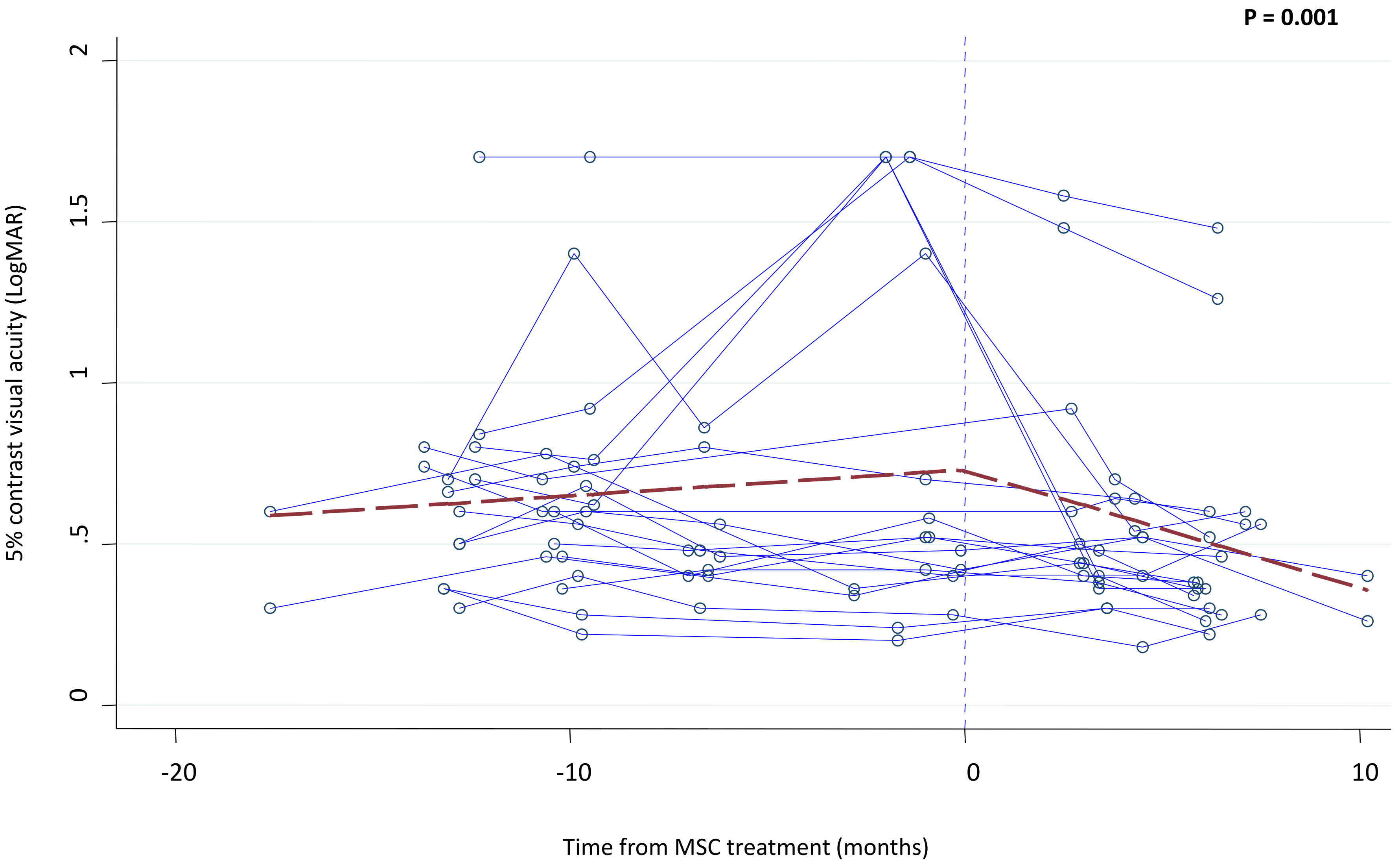

Change in 5% contrast visual acuity is shown for individual patients before and after treatment connected by solid lines. Pre- and post-treatment average rates of change are also shown connected in red dash. The significance test are shown is for a change in the gradient at the time of intervention.

Supplementary figure 2D: change in 1.25% contrast acuity over time

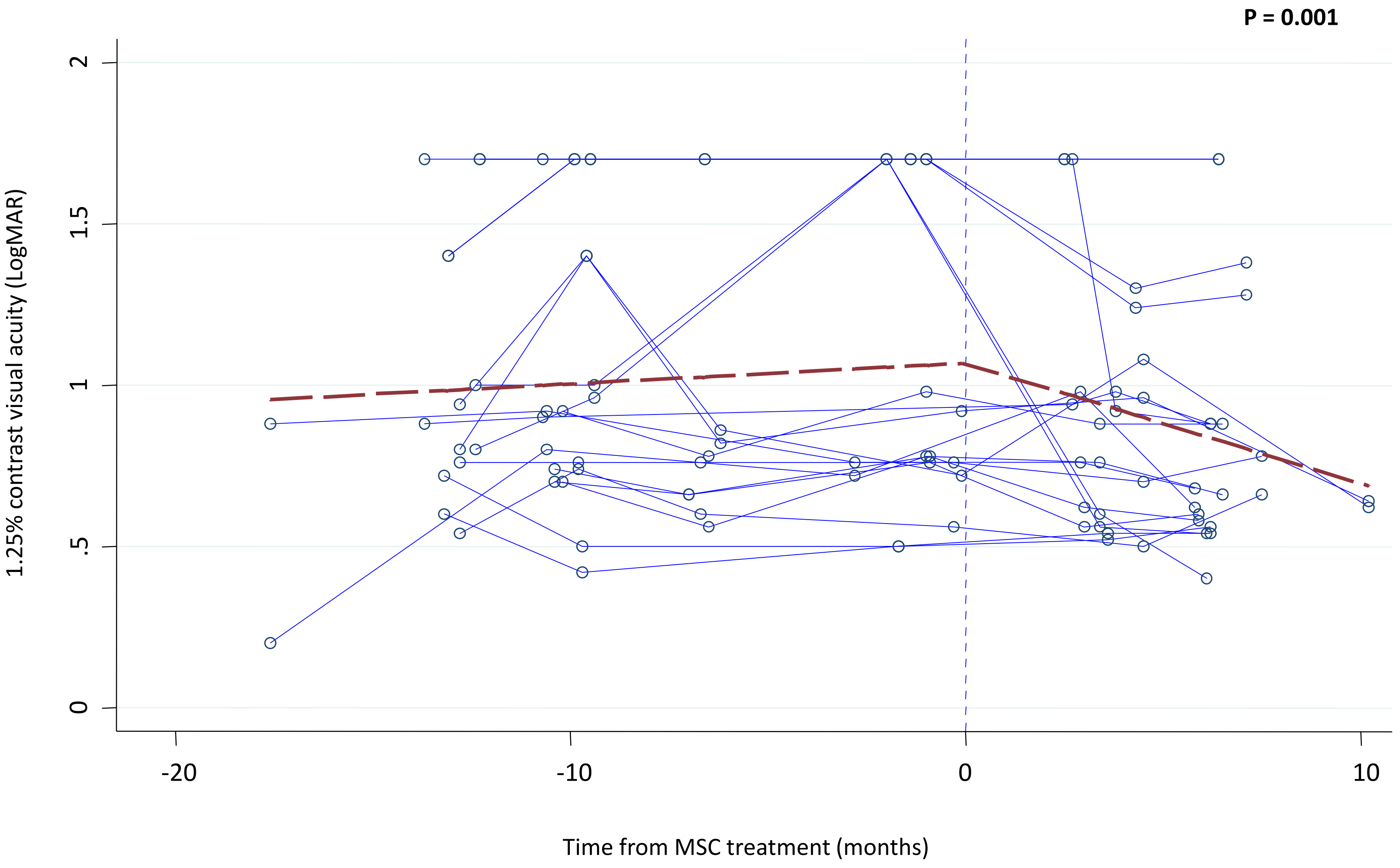

Change in 1.25% contrast visual acuity is shown for individual patients before and after treatment connected by solid lines. Pre- and post-treatment average rates of change are also shown connected in red dash. The significance test are shown is for a change in the gradient at the time of intervention.

Supplementary figure 2E: change in colour vision over time

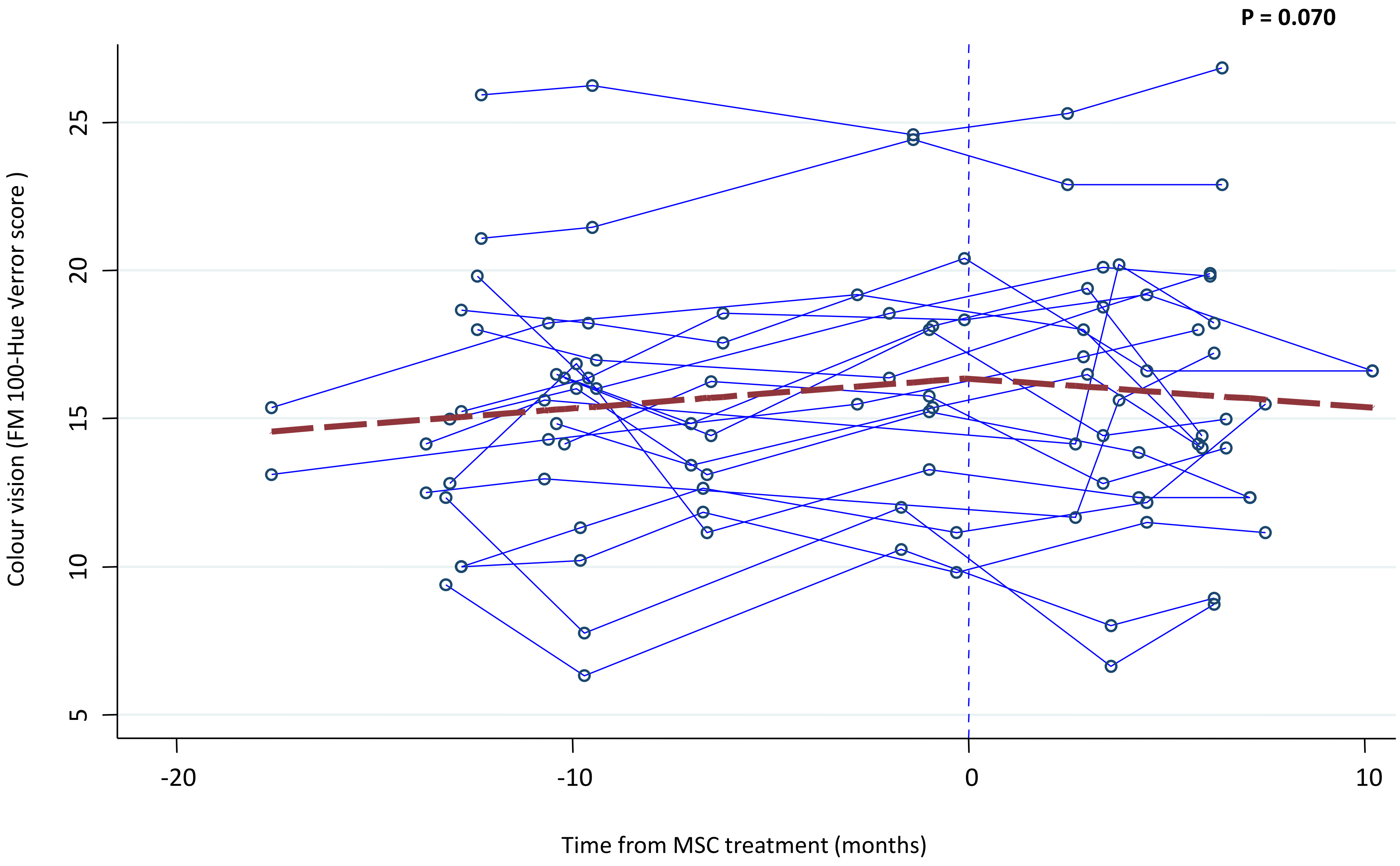

Change in colour vision (square root total error score) is shown for individual patients before and after treatment connected by solid lines. Pre- and post-treatment average rates of change are also shown connected in red dash. The significance test are shown is for a change in the gradient at the time of intervention.

Supplementary figure 2F: change in visual field over time

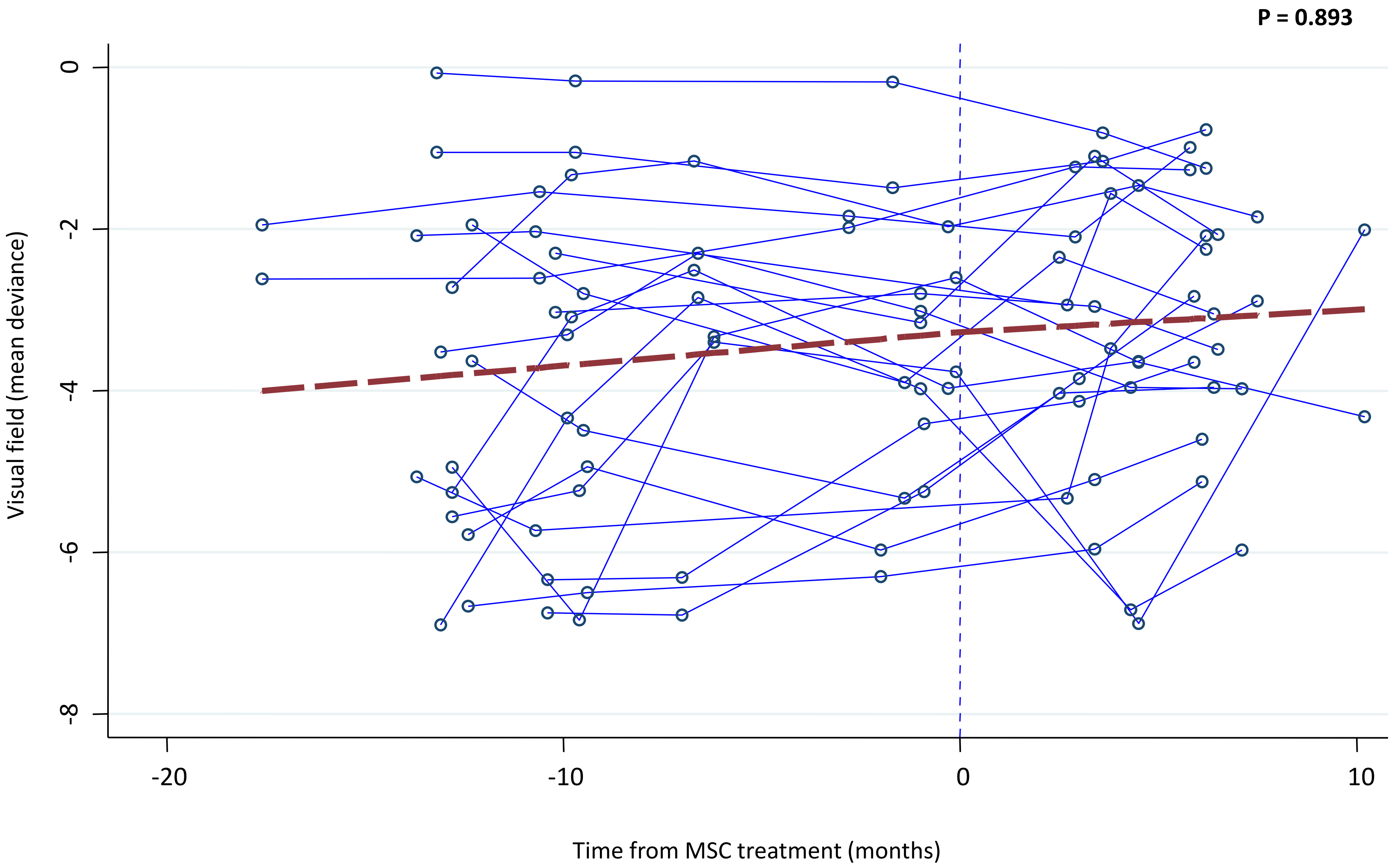

Change in 25% contrast visual acuity is shown for individual patients before and after treatment connected by solid lines. Pre- and post-treatment average rates of change are also shown connected in red dash. The significance test are shown is for a change in the gradient at the time of intervention.

Supplementary figure 2G: change in visual evoked response (VER) latency over time

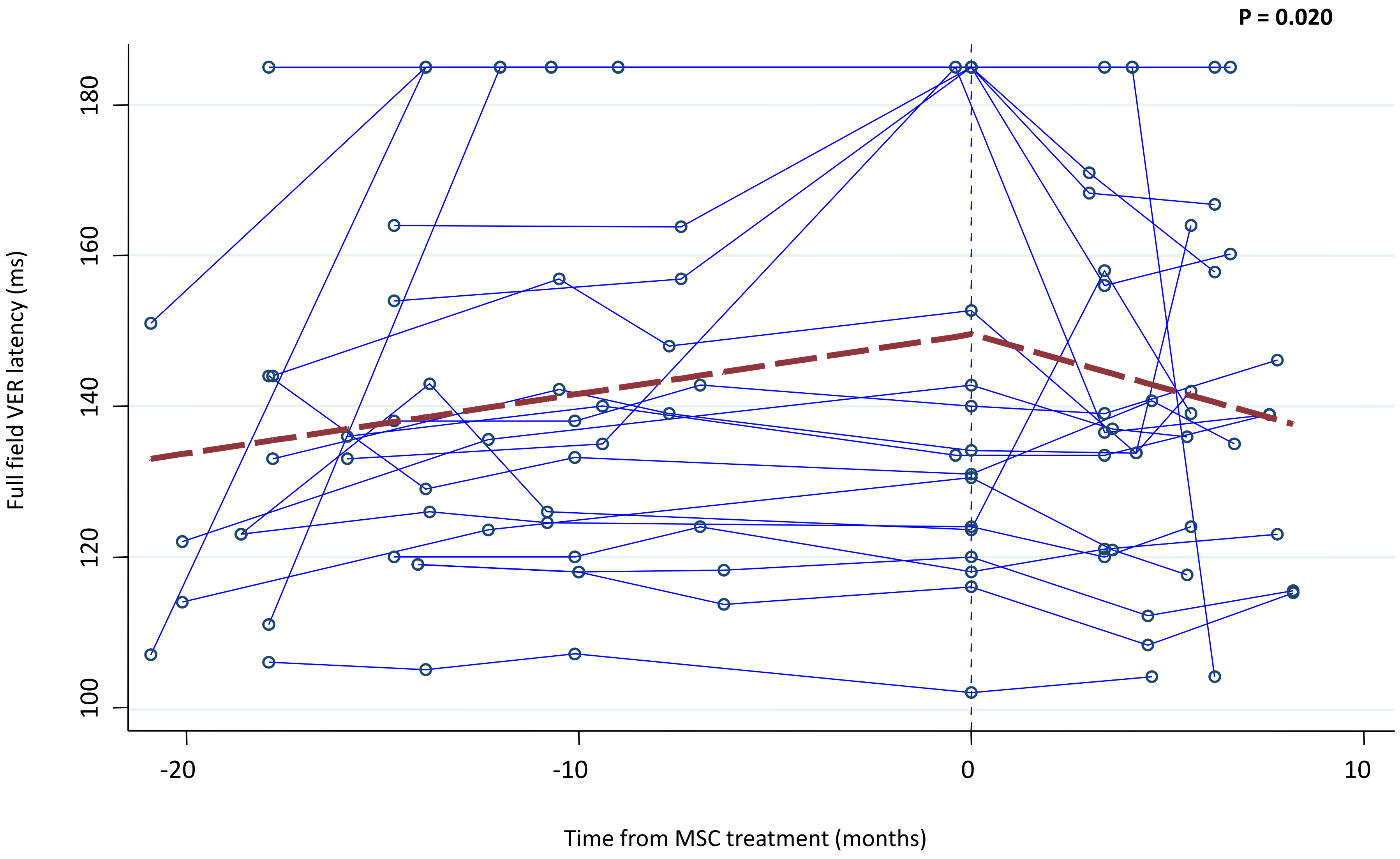

Change in full field visual evoked response latency is shown for individual patients before and after treatment connected by solid lines. Pre- and post-treatment average rates of change are also shown connected in red dash. The significance test are shown is for a change in the gradient at the time of intervention.

Supplementary figure 2H: change in visual evoked response (VER) amplitude over time

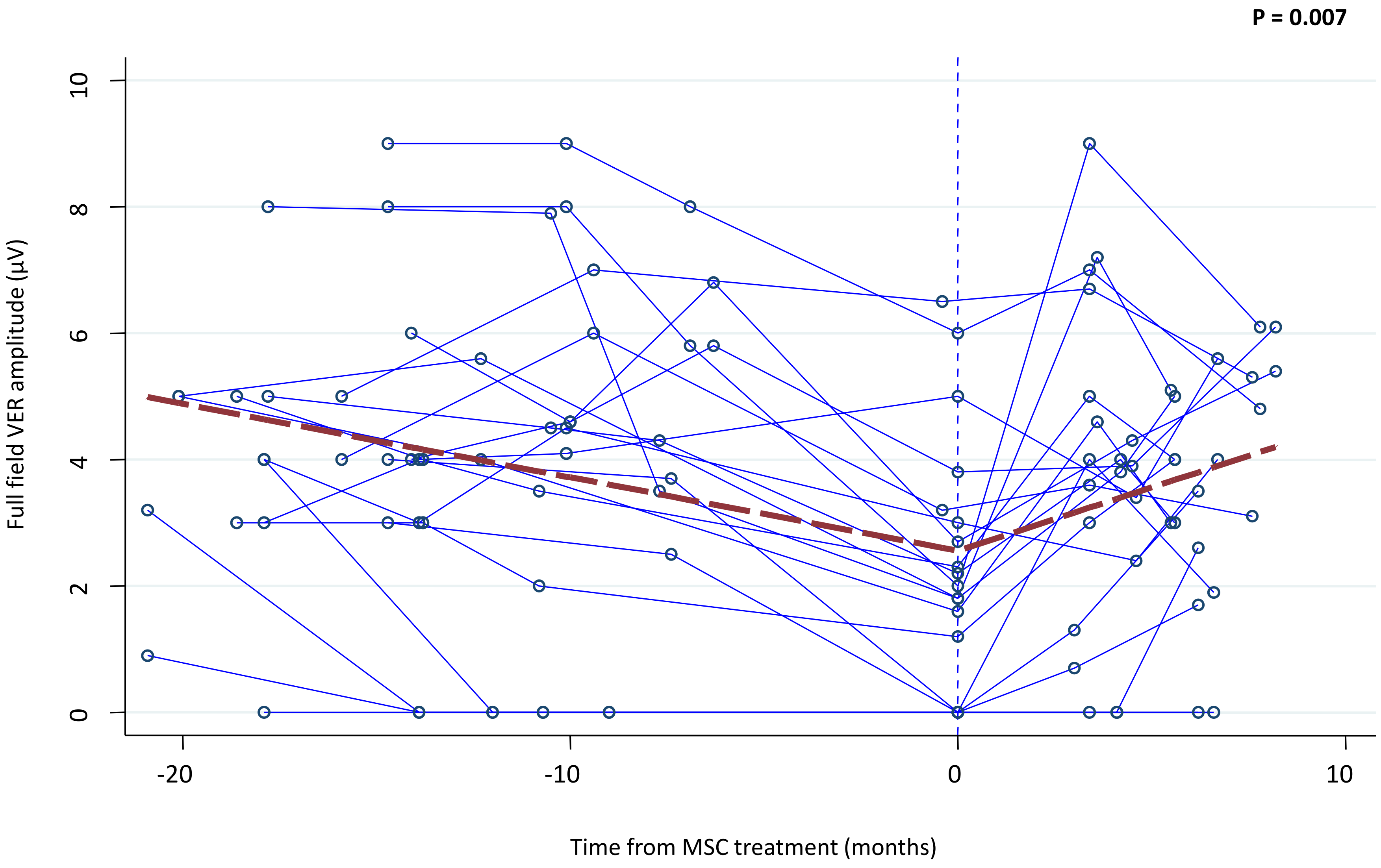

Change in full field visual evoked response amplitude is shown for individual patients before and after treatment connected by solid lines. Pre- and post-treatment average rates of change are also shown connected in red dash. The significance test are shown is for a change in the gradient at the time of intervention.

Supplementary figure 2I: change in macular volume over time

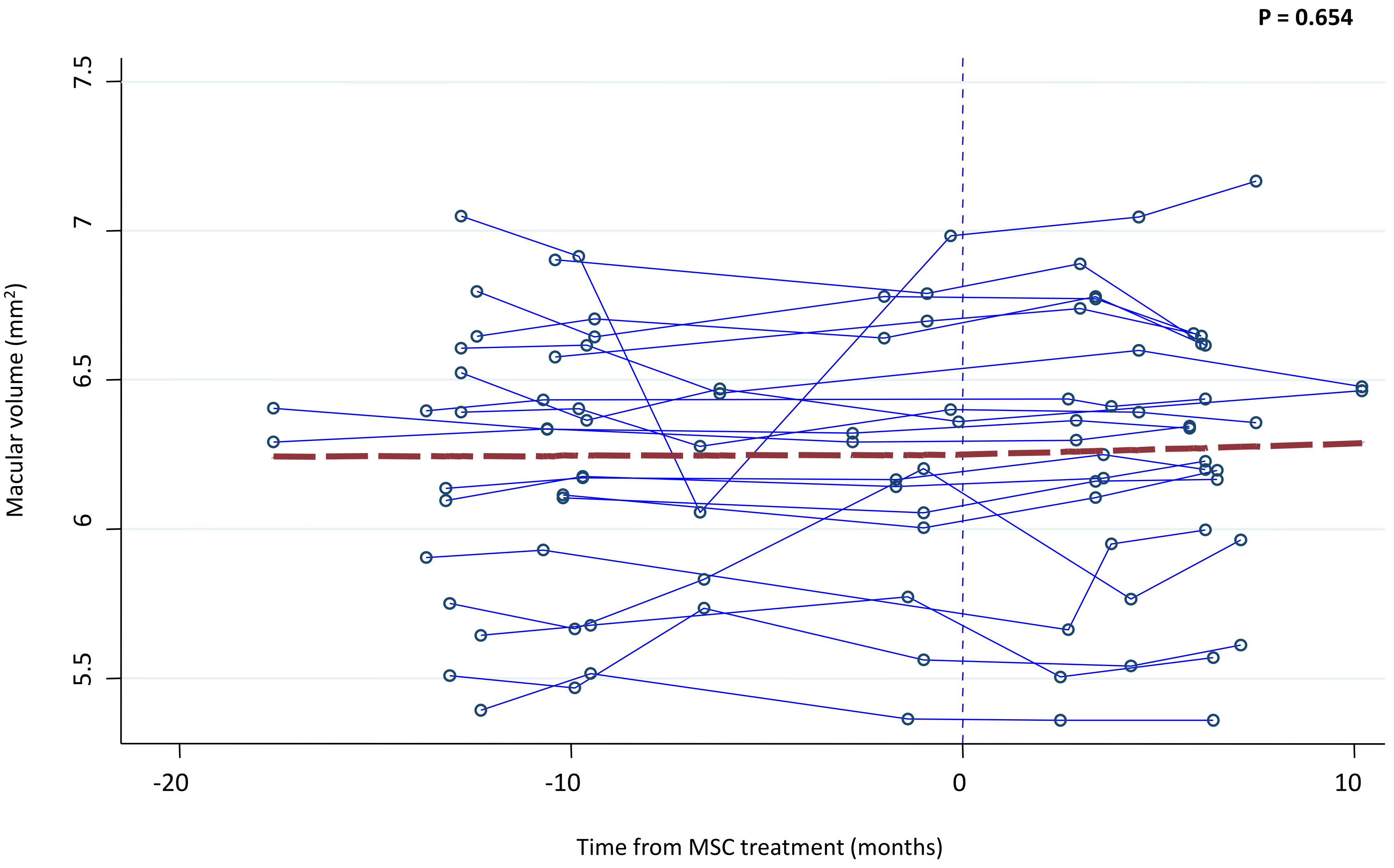

Change in macular volume is shown for individual patients before and after treatment connected by solid lines. Pre- and post-treatment average rates of change are also shown connected in red dash. The significance test are shown is for a change in the gradient at the time of intervention.

Supplementary figure 2J: change in retinal nerve fibre layer thickness over time

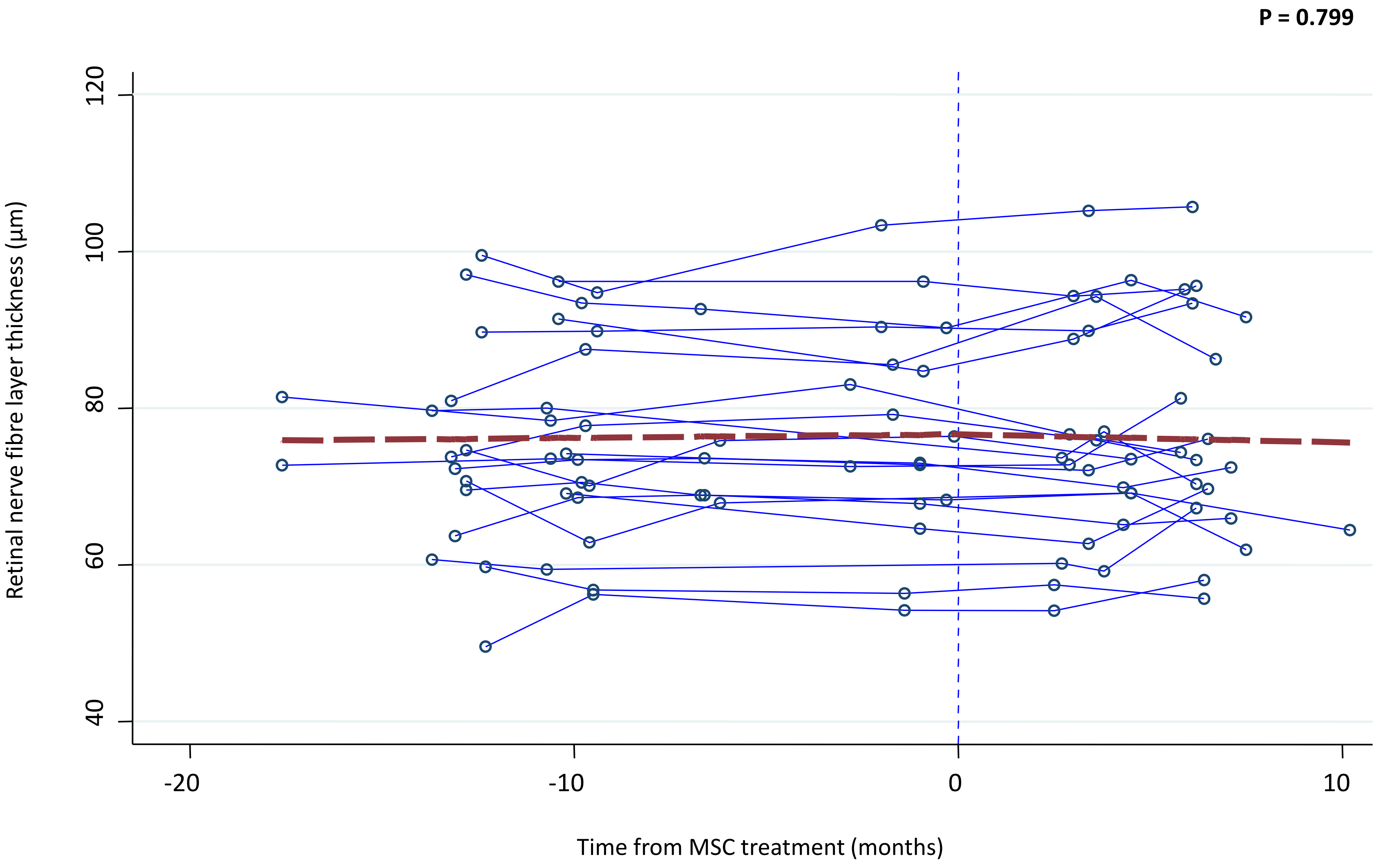

Change in retinal nerve fibre layer thickness is shown for individual patients before and after treatment connected by solid lines. Pre- and post-treatment average rates of change are also shown connected in red dash. The significance test are shown is for a change in the gradient at the time of intervention.

Supplementary figure 2K: change in optic nerve area over time

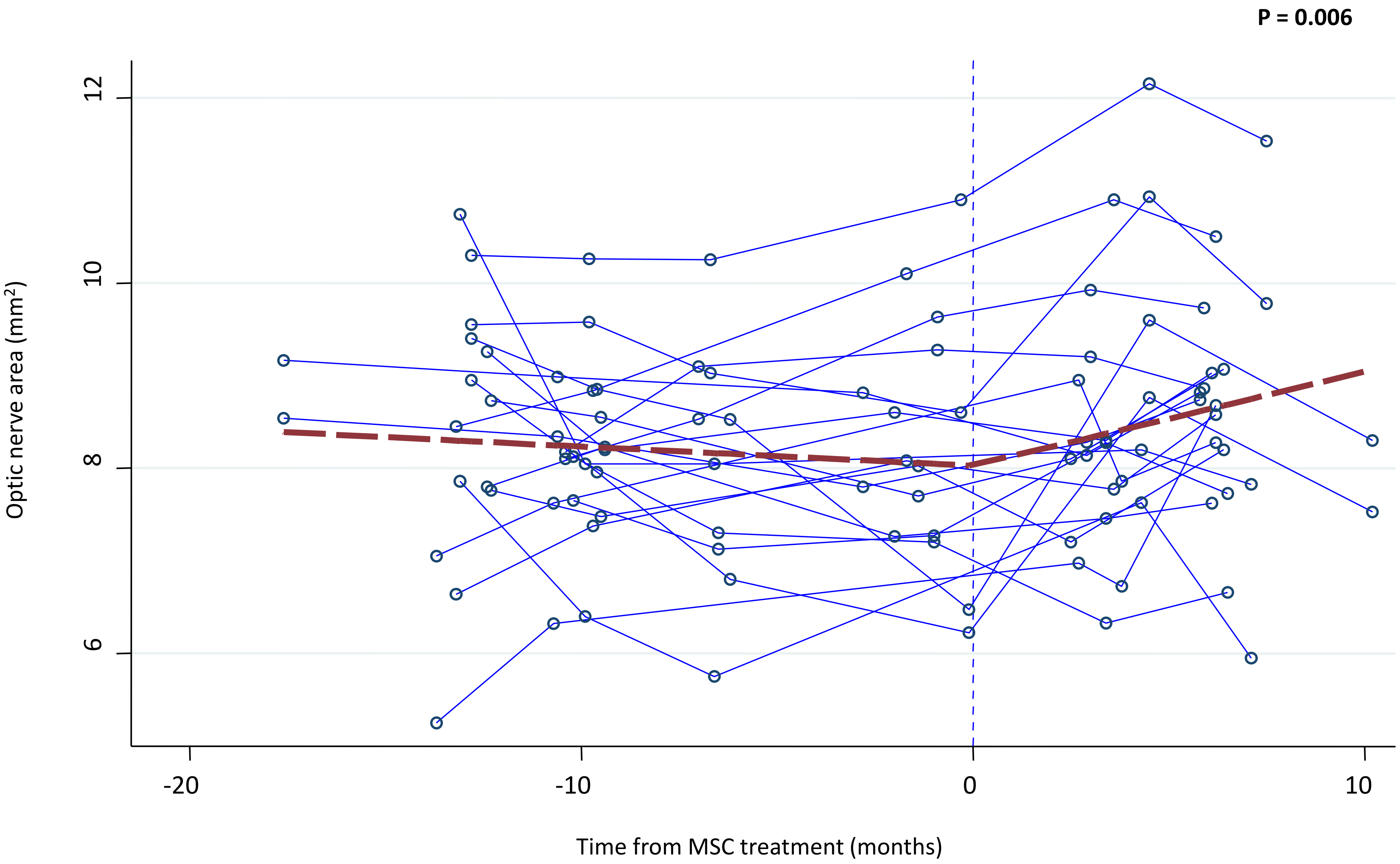

Change in optic nerve area is shown for individual patients before and after treatment connected by solid lines. Pre- and post-treatment average rates of change are also shown connected in red dash. The significance test are shown is for a change in the gradient at the time of intervention.

Supplementary figure 2L: change in optic nerve magnetization transfer ratio (MTR) over time

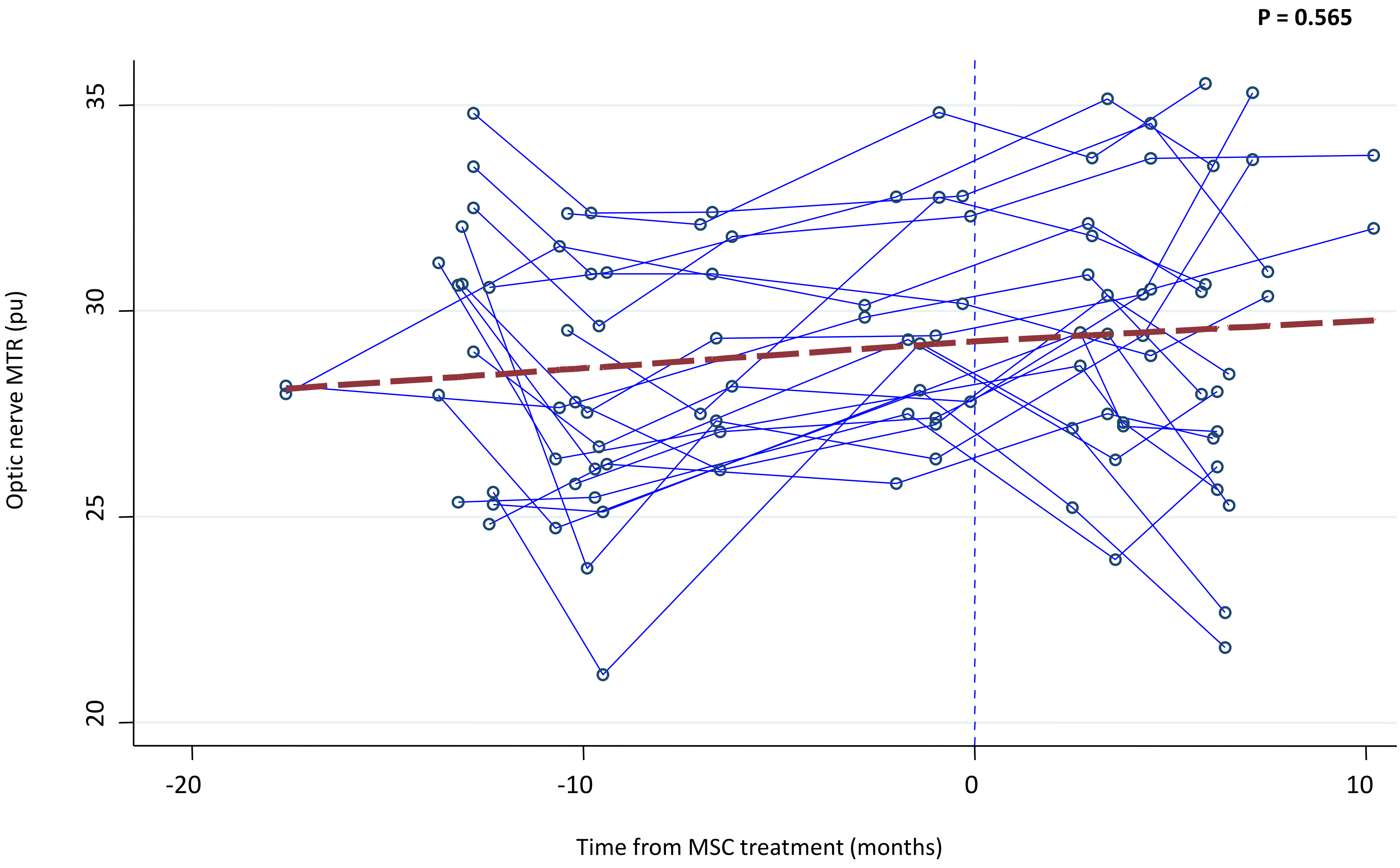

Change in optic nerve magnetization transfer ratio is shown for individual patients before and after treatment connected by solid lines. Pre- and post-treatment average rates of change are also shown connected in red dash. The significance test are shown is for a change in the gradient at the time of intervention.

Supplementary figure 2M: change in expanded disability status scale (EDSS) over time

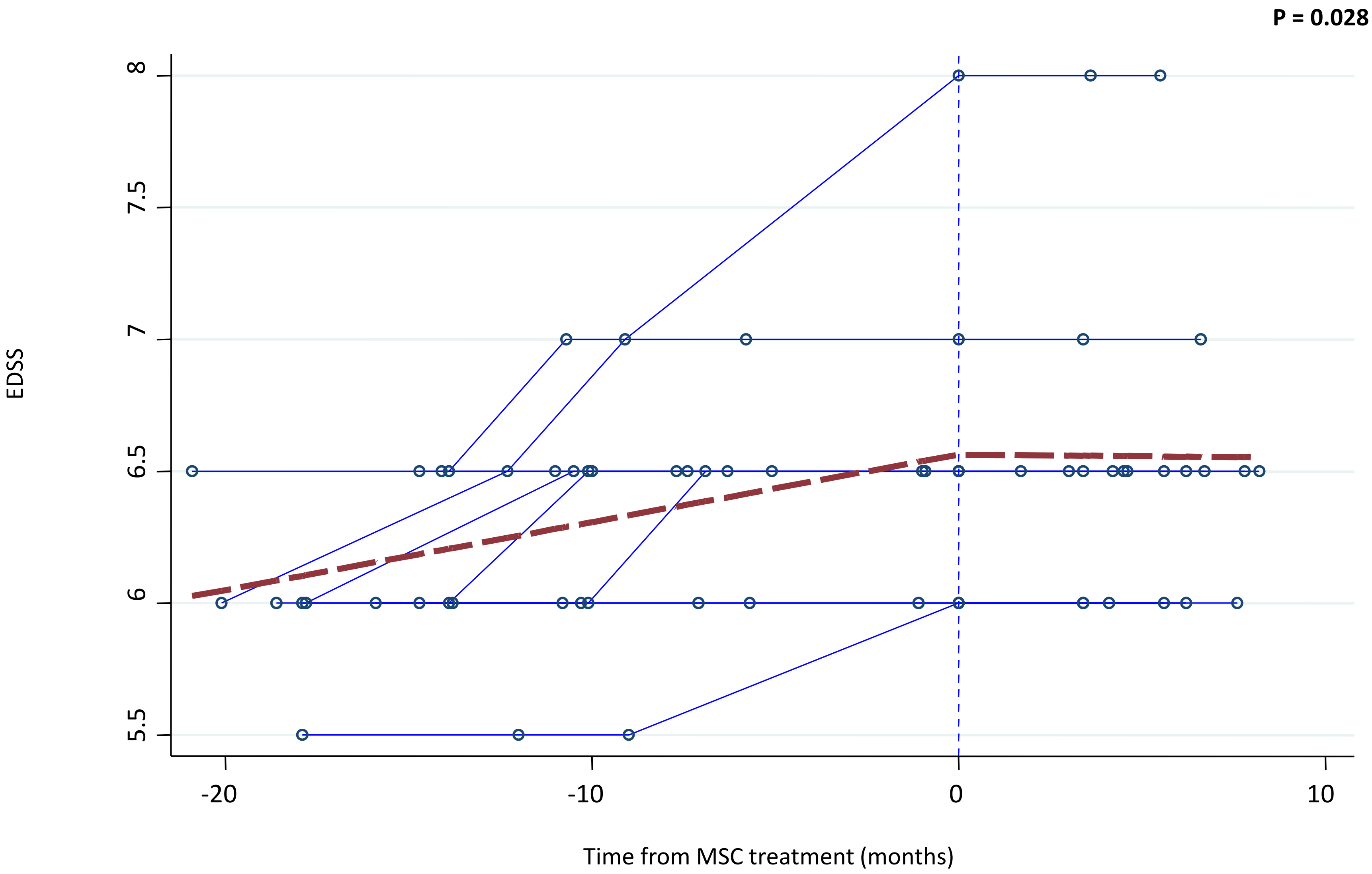

Change in expanded disability status scale (EDSS) is shown for individual patients before and after treatment connected by solid lines. Pre- and post-treatment average rates of change are also shown connected in red dash. The significance test are shown is for a change in the gradient at the time of intervention.

Supplementary figure 2N: change in multiple sclerosis functional composite (MSFC) over time

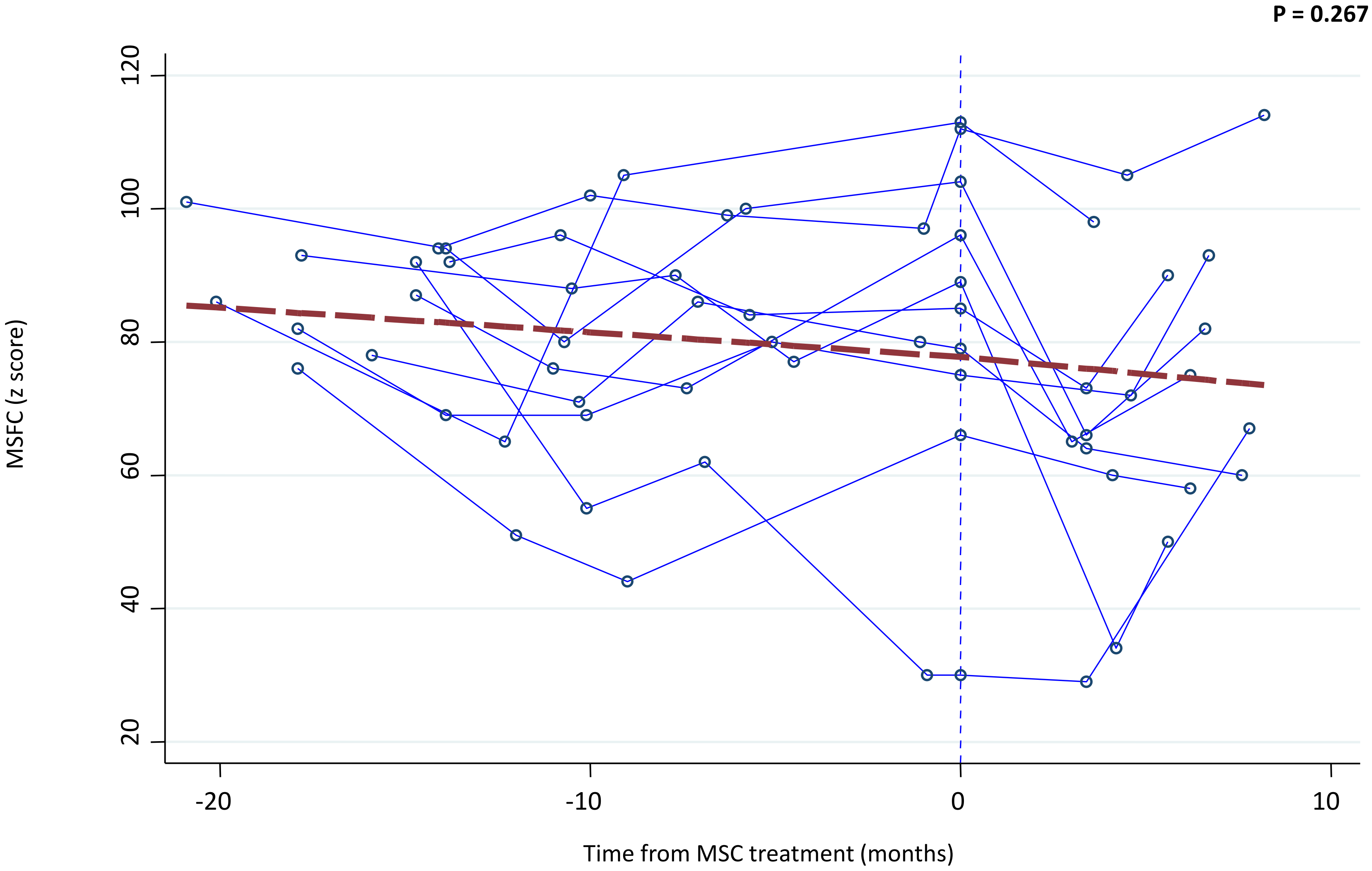

Change in multiple sclerosis functional composite is shown for individual patients before and after treatment connected by solid lines. Pre- and post-treatment average rates of change are also shown connected in red dash. The significance test are shown is for a change in the gradient at the time of intervention.

Supplementary figure 2O: change in Addenbrooke's cognitive examination revised (ACE-R) over time

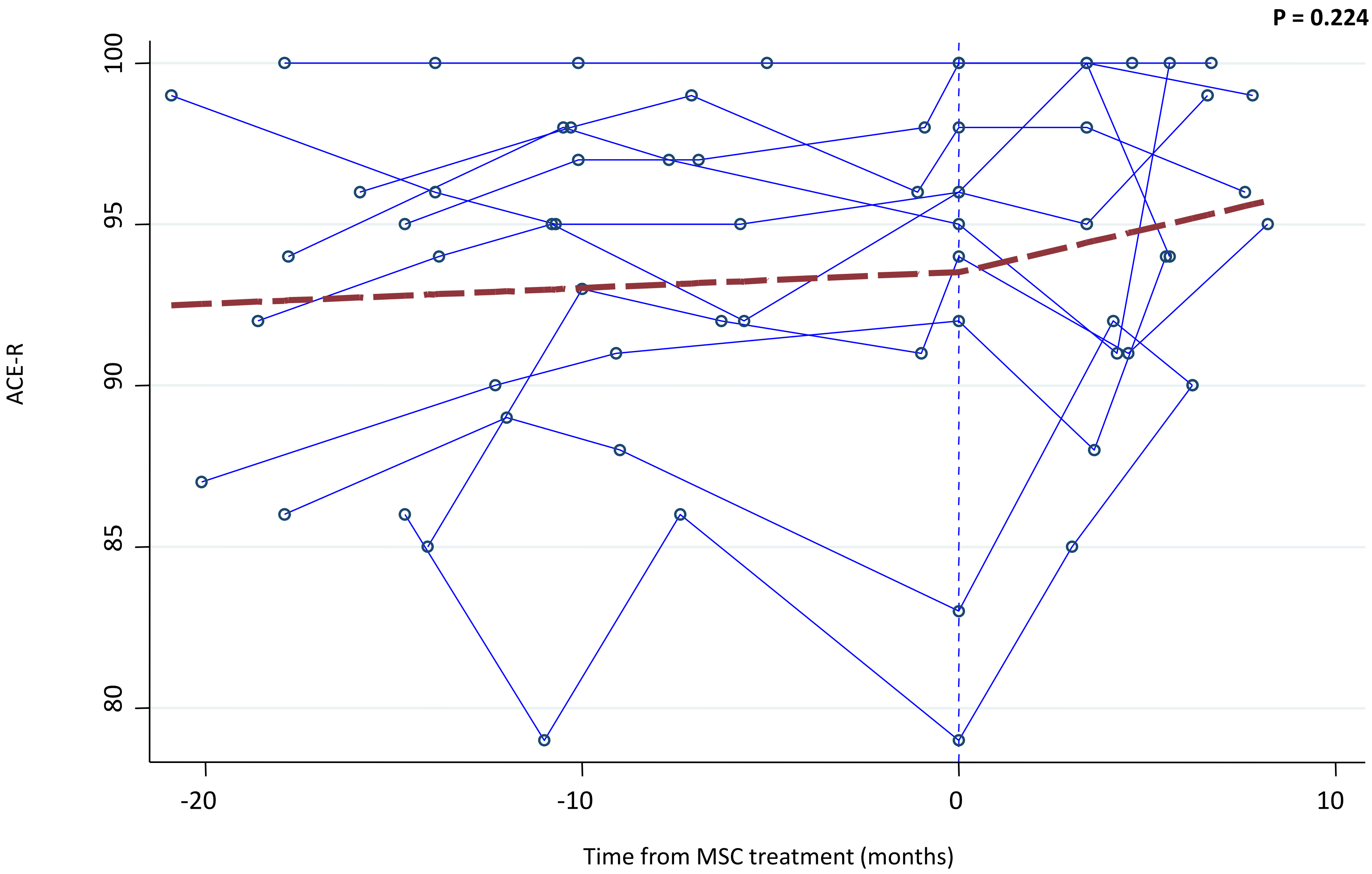

Change in Addenbrooke's cognitive examination-revised score is shown for individual patients before and after treatment connected by solid lines. Pre- and post-treatment average rates of change are also shown connected in red dash. The significance test are shown is for a change in the gradient at the time of intervention.

Supplementary figure 2P: change in multiple sclerosis impact scale over time

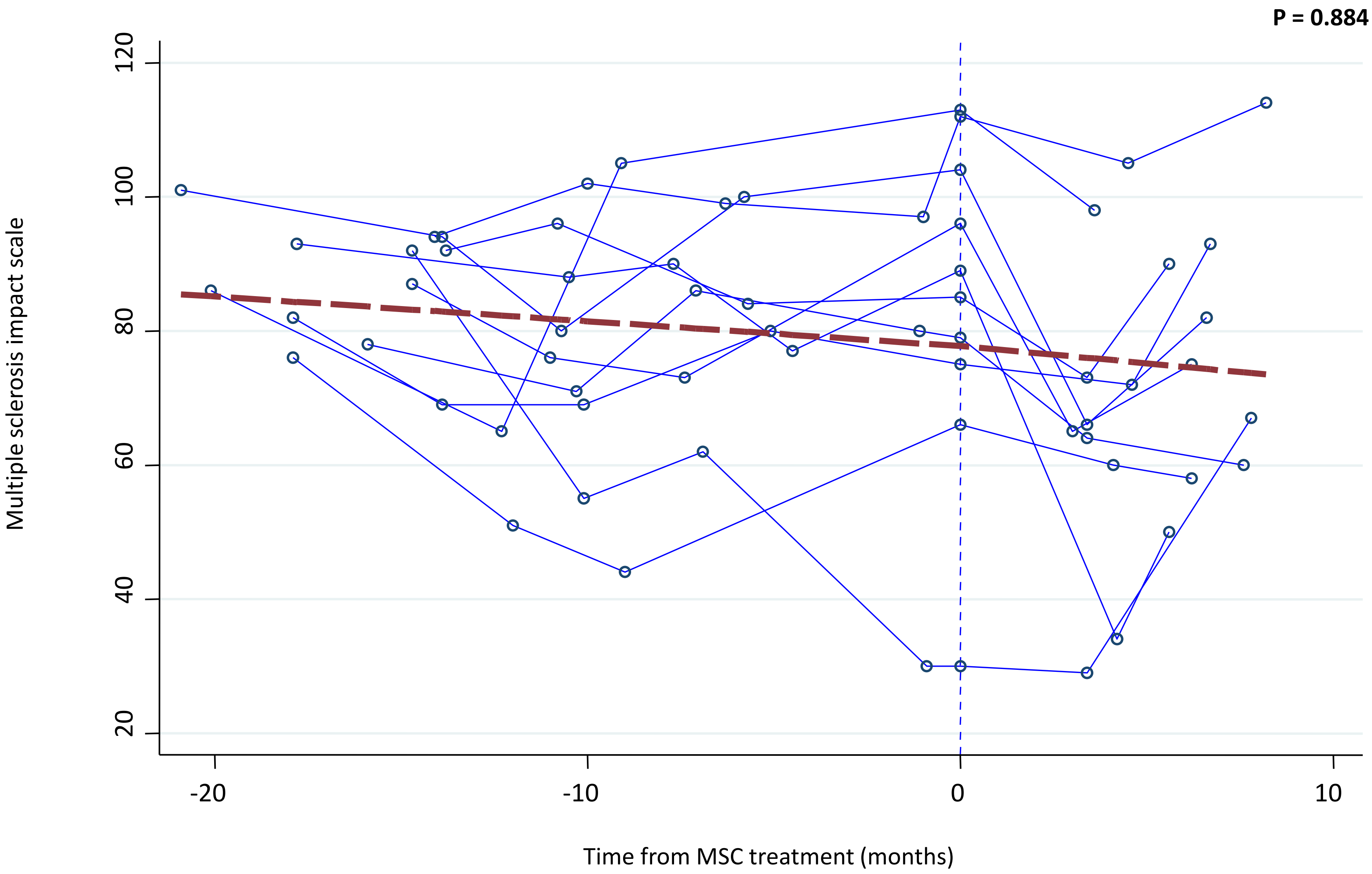

Change in 29-item multiple sclerosis impact scale is shown for individual patients before and after treatment connected by solid lines. Pre- and post-treatment average rates of change are also shown connected in red dash. The significance test are shown is for a change in the gradient at the time of intervention.

Supplementary figure 2Q: change in Beck depression inventory over time

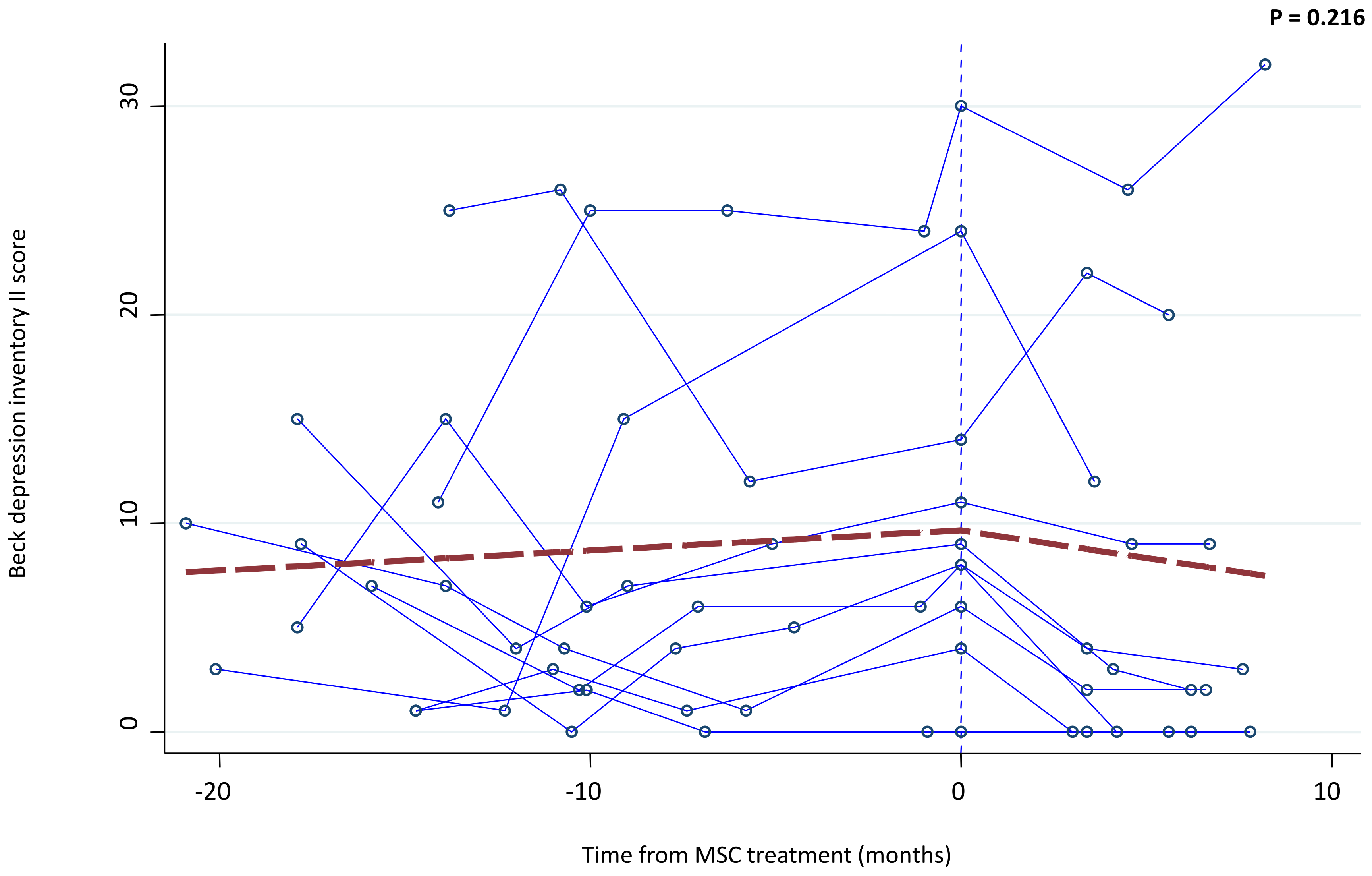

Change in Beck depression inventory II score is shown for individual patients before and after treatment connected by solid lines. Pre- and post-treatment average rates of change are also shown connected in red dash. The significance test are shown is for a change in the gradient at the time of intervention.

Supplementary figure 2R: change in total brain T1 hypointense lesion volume over time

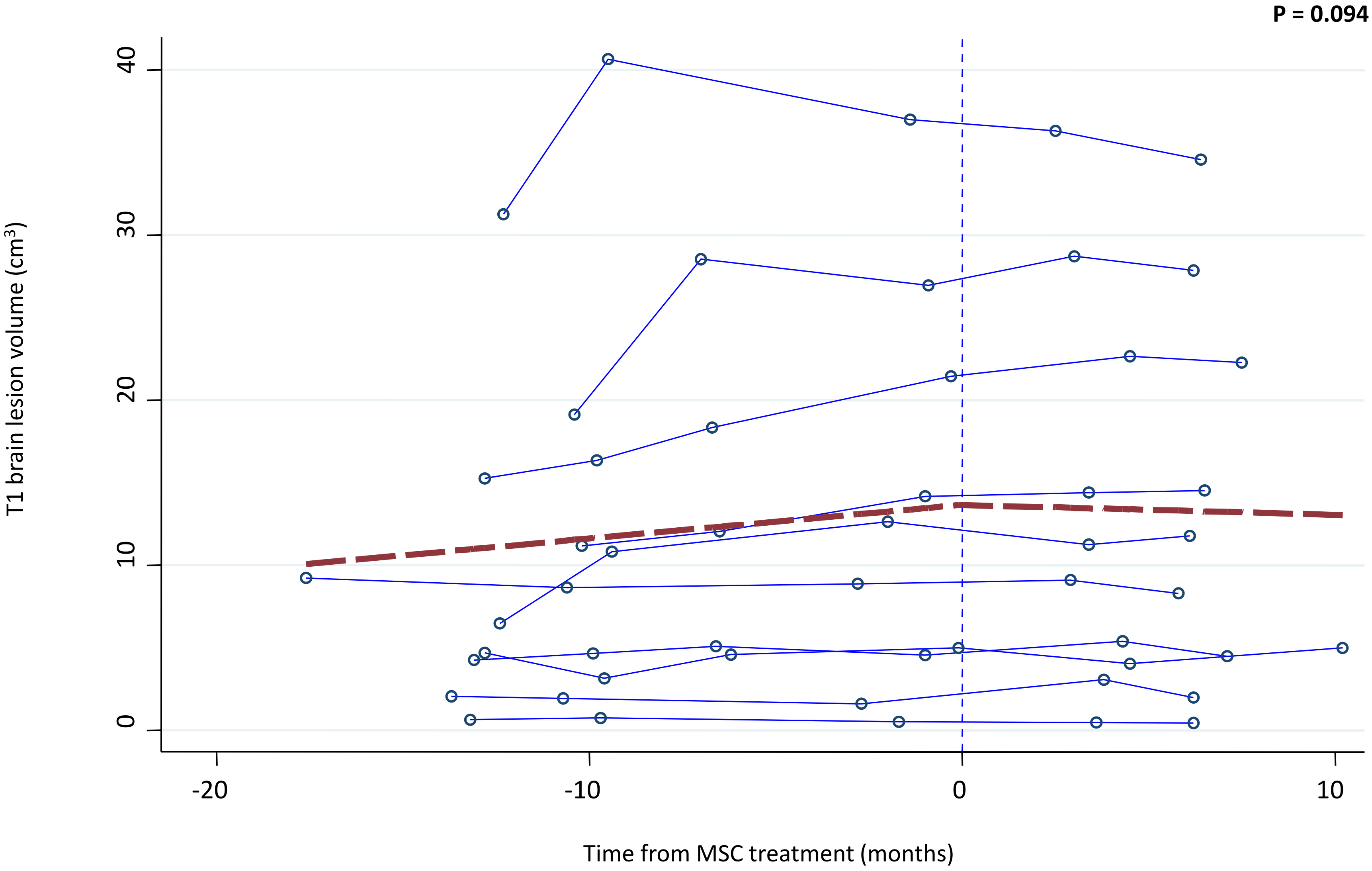

Change in T1 brain lesion volume is shown for individual patients before and after treatment connected by solid lines. Pre- and post-treatment average rates of change are also shown connected in red dash. The significance test are shown is for a change in the gradient at the time of intervention.

Supplementary figure 2S: change in total brain T1 hypointense lesion magnetization transfer ratio (MTR) over time

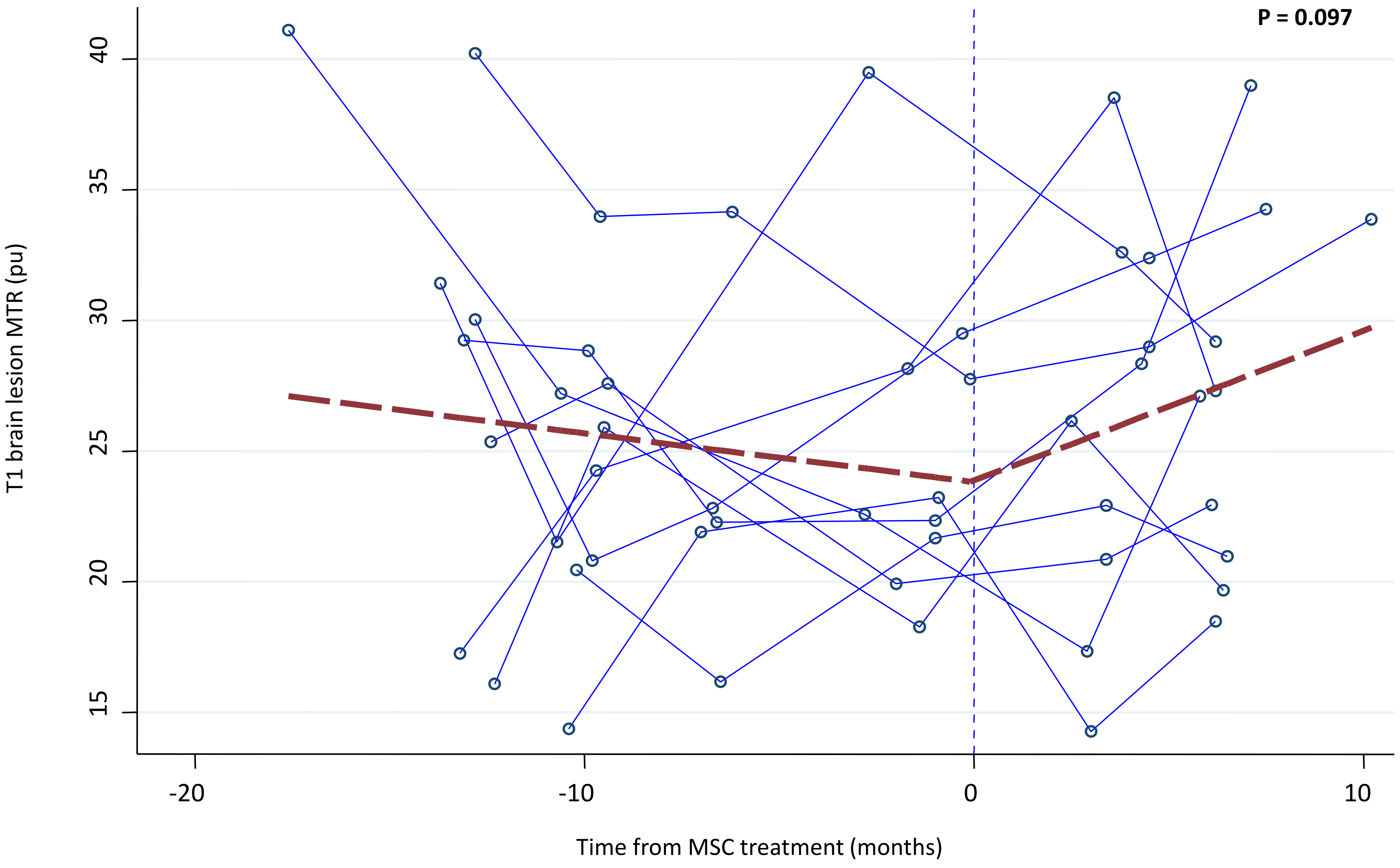

Change in T1 brain lesion magnetization transfer ratio is shown for individual patients before and after treatment connected by solid lines. Pre- and post-treatment average rates of change are also shown connected in red dash. The significance test are shown is for a change in the gradient at the time of intervention.

Supplementary figure 2T: change in total brain T2 intense lesion volume over time

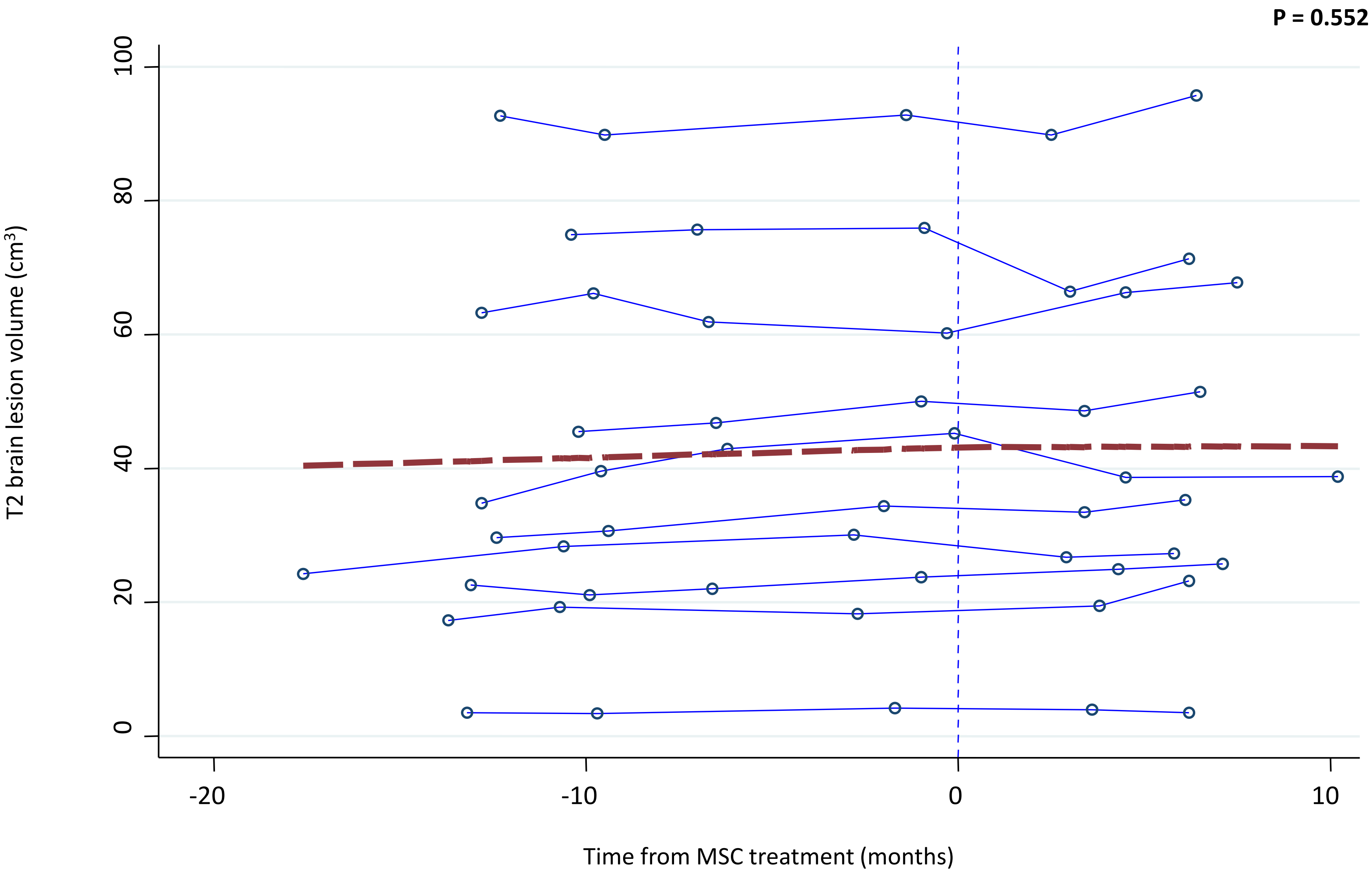

Change in T2 brain lesion volume is shown for individual patients before and after treatment connected by solid lines. Pre- and post-treatment average rates of change are also shown connected in red dash. The significance test are shown is for a change in the gradient at the time of intervention.

Supplementary figure 2U: change in total brain T2 intense lesion magnetization transfer ratio (MTR) over time

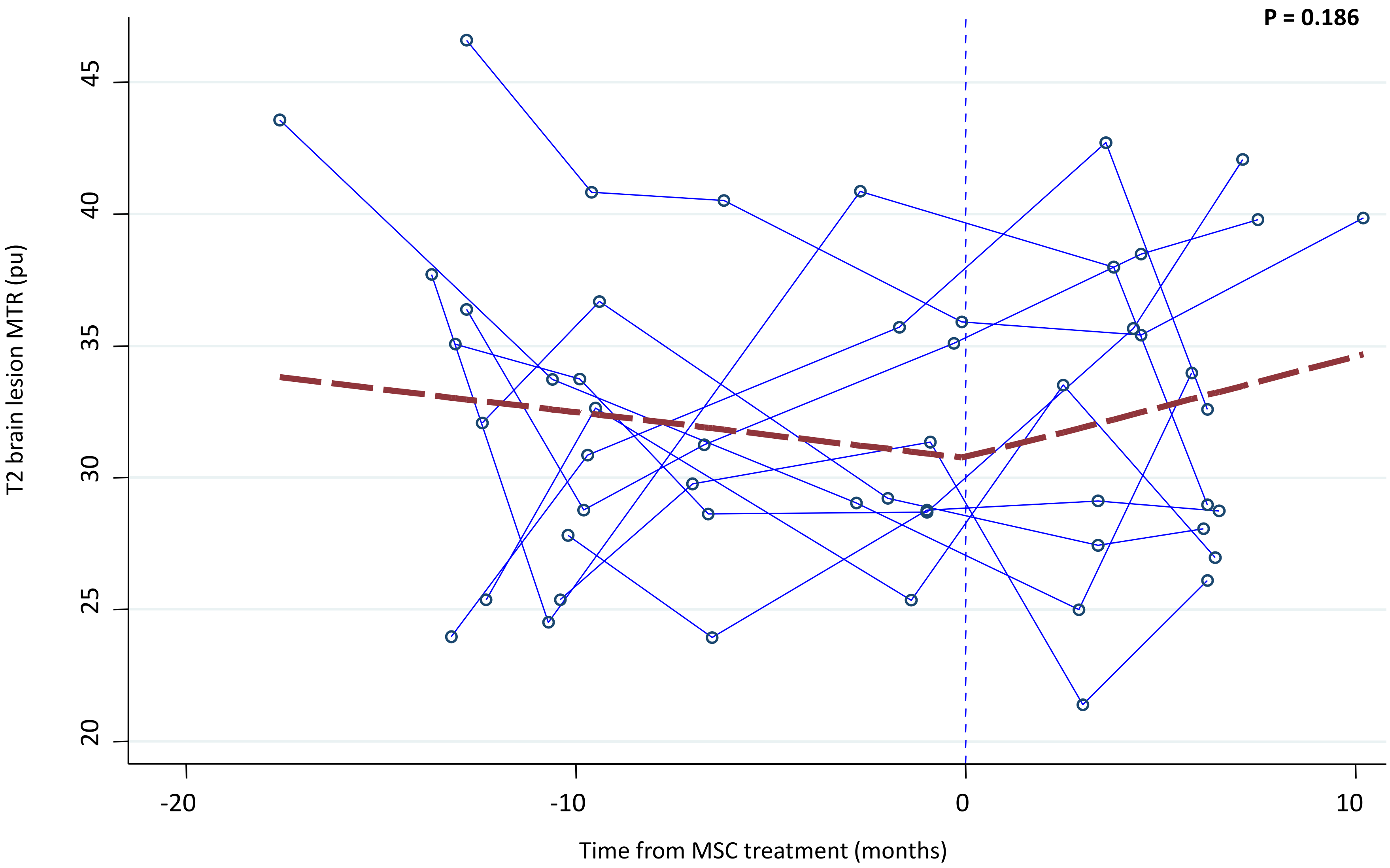

Change in T2 brain lesion magnetization transfer ratio is shown for individual patients before and after treatment connected by solid lines. Pre- and post-treatment average rates of change are also shown connected in red dash. The significance test are shown is for a change in the gradient at the time of intervention.
